# Glutathionylation of *Yersinia pestis* LcrV and Its Effects on Plague Pathogenesis

**DOI:** 10.1128/mBio.00646-17

**Published:** 2017-05-16

**Authors:** Anthony Mitchell, Christina Tam, Derek Elli, Thomas Charlton, Patrick Osei-Owusu, Farbod Fazlollahi, Kym F. Faull, Olaf Schneewind

**Affiliations:** a Howard T. Ricketts Laboratory, Argonne National Laboratory, Lemont, lllinois, USA; b Department of Microbiology, University of Chicago, Chicago, Illinois, USA; c Mass Spectrometry Laboratory, Department of Psychiatry and Biobehavioral Sciences, Semel Institute of Neuroscience and Human Behavior, David Geffen School of Medicine, University of California, Los Angeles, California, USA; University of Washington

**Keywords:** macrophage, *Yersinia pestis*, glutathionylation, innate immunity, plague, ribosomal protein S3 (RPS3)

## Abstract

Glutathionylation, the formation of reversible mixed disulfides between glutathione and protein cysteine residues, is a posttranslational modification previously observed for intracellular proteins of bacteria. Here we show that *Yersinia pestis* LcrV, a secreted protein capping the type III secretion machine, is glutathionylated at Cys^273^ and that this modification promotes association with host ribosomal protein S3 (RPS3), moderates *Y. pestis* type III effector transport and killing of macrophages, and enhances bubonic plague pathogenesis in mice and rats. Secreted LcrV was purified and analyzed by mass spectrometry to reveal glutathionylation, a modification that is abolished by the codon substitution Cys^273^Ala in *lcrV*. Moreover, the *lcrV*_*C273A*_ mutation enhanced the survival of animals in models of bubonic plague. Investigating the molecular mechanism responsible for these virulence attributes, we identified macrophage RPS3 as a ligand of LcrV, an association that is perturbed by the Cys^273^Ala substitution. Furthermore, macrophages infected by the *lcrV*_*C273A*_ variant displayed accelerated apoptotic death and diminished proinflammatory cytokine release. Deletion of *gshB*, which encodes glutathione synthetase of *Y. pestis*, resulted in undetectable levels of intracellular glutathione, and we used a *Y. pestis* Δ*gshB* mutant to characterize the biochemical pathway of LcrV glutathionylation, establishing that LcrV is modified after its transport to the type III needle via disulfide bond formation with extracellular oxidized glutathione.

## IMPORTANCE

*Yersinia pestis*, the causative agent of plague, has killed large segments of the human population; however, the molecular bases for the extraordinary virulence attributes of this pathogen are not well understood. We show here that LcrV, the cap protein of bacterial type III secretion needles, is modified by host glutathione and that this modification contributes to the high virulence of *Y. pestis* in mouse and rat models for bubonic plague. These data suggest that *Y. pestis* exploits glutathione in host tissues to activate a virulence strategy, thereby accelerating plague pathogenesis.

## INTRODUCTION

Glutathione, the predominant low-molecular-weight (LMW) peptide thiol present in all mitochondrial eukaryotes and nearly all Gram-negative bacteria, exists as a redox couple between its reduced (GSH) and oxidized (GSSG) forms and plays a pivotal role in many physiological processes (1). In *Escherichia coli*, glutathione maintains the proper oxidation state of protein thiols, regulates intracellular potassium levels, detoxifies methylglyoxal and halogenated compounds, and protects bacteria against acidic, oxidative, and osmotic stresses (2). Protein glutathionylation, a posttranslational modification whereby the sulfhydryl of glutathione forms a reversible mixed disulfide with protein cysteine residues, is increased during oxidative stress and protects reactive protein thiols from potentially irreversible oxidation (3). Glutathione in body fluids of vertebrates has been shown to impact the pathogenesis of bacterial infections by regulating the expression of microbial virulence functions or by protecting against oxidative and nitrosative stresses (4–6). A role for extracellular glutathione in modifying the surface of bacterial pathogens has heretofore not been described.

*Yersinia pestis*, the etiologic agent of plague, has killed more people worldwide than any other infectious agent (7). This bacterium employs a specialized type III secretion system to evade and neutralize the immune responses of its mammalian hosts (8). Particularly significant in this respect is the multifunctional virulence factor LcrV, the plague-protective antigen and needle cap protein of the *Yersinia* type III secretion machine (9, 10). LcrV is essential for plague pathogenesis (11) as the polypeptide enables the transport of *Yersinia* effector proteins (Yops) across the eukaryotic plasma membrane, where they exert cytotoxic and immunomodulatory effects within host immune cells (12). Here we report that *Y. pestis* LcrV is glutathionylated at cysteine residue 273 and that this modification impacts the pathogenesis of plague infections.

## RESULTS AND DISCUSSION

### LcrV, the cap protein of *Y. pestis* type III secretion machines, is glutathionylated at Cys^273^.

**FIG 1:**
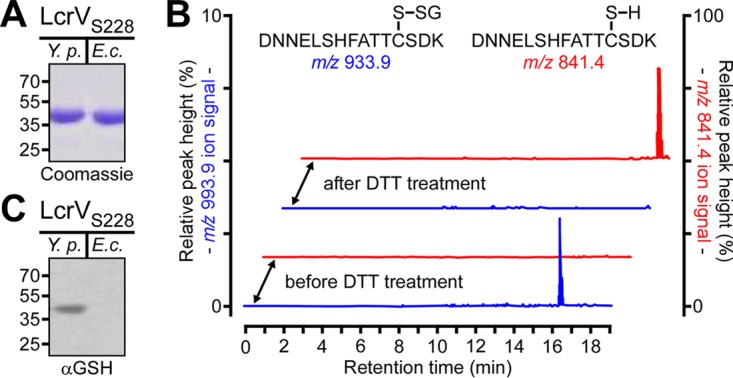
LcrV secreted by *Y. pestis* is glutathionylated at Cys^273^. (A) Coomassie-stained SDS-PAGE of LcrV_S228_ purified via Strep-Tactin affinity chromatography from either the culture supernatant of *Y. pestis* KLD29(pKG48) (*Y.p.*) or cell lysates of *E. coli* DH5α(pKG48) (*E.c.*). (B) Reconstructed ion traces for the unmodified (*m/z* 841.4) [(M+2H)^2+^ ion of 1,680.71] and glutathione-modified (*m/z* 993.9) [(M+2H)^2+^ ion of 1,985.79] tryptic peptides from the *Y. pestis* LcrV_S228_ protein encompassing Cys^273^ before and after treatment with dithiothreitol (DTT). The chromatograms show the absence of the unmodified peptides and the presence of the glutathione-modified peptides before treatment and following DTT treatment, the presence of the unmodified peptides, and the absence of the glutathione-modified peptides. (C) LcrV_S228_ purified from *Y. pestis* culture supernatants or *E. coli* extracts (rLcrV_S228_) was analyzed by immunoblotting with glutathione-specific antiserum (αGSH).

LcrV_S228_ carries a Strep-tag insertion at amino acid 228 that does not affect its function (13). LcrV_S228_ was purified from the culture supernatant of *Y. pestis* KLD29 Δ*lcrV*(pKG48), and the recombinant form of LcrV_S228_ (rLcrV_S228_) was purified from extracts of *E. coli* DH5α(pKG48). Both proteins were analyzed by combined liquid chromatography-electrospray ionization mass spectrometry (LC-ESI-MS) to reveal average masses of 38,594.4 Da (LcrV_S228_) and 38,284.9 Da (rLcrV_S228_) (Fig. 1A; see Fig. S1A and B in the supplemental material). *Y. pestis* LcrV_S228_ was 309.5 Da heavier than *E. coli* rLcrV_S228_ and 314.2 Da heavier than the calculated mass for the 334-amino-acid protein with the initiator methionine (38,280.22 Da). Edman degradation of *Y. pestis* LcrV_S228_ released N-terminal amino acids with the sequence NH_2_-MIRA-CO_2_H, identical to the predicted protein sequence (11) (Fig. S1C). To locate the site of the modification, reverse-phase-purified *Y. pestis* LcrV_S228_ and *E. coli* rLcrV_S228_ were treated with trypsin, and overlapping cleavage maps from LC-ESI-MS data were compared (see Table S1 in the supplemental material). These experiments identified peptide 270-284 (NH_2_-DNNELSHFATTCSDK-CO_2_H) as the site of the modification in *Y. pestis* LcrV_S228_ because its measured monoisotopic mass (1,985.79 Da) was 305.08 Da heavier than that obtained for rLcrV_S228_ (1,680.71 Da), which is in close agreement with the calculated mass for the predicted peptide (1,680.70 Da) (Table S1). Collisionally induced dissociation of the 1,985.79-Da LcrV_S228_ peptide generated fragment ions that identified Cys^273^ as the site of modification (see Table S2 in the supplemental material). Treatment with dithiothreitol (DTT), a disulfide reductant, collapsed the average mass of *Y. pestis* LcrV_S228_ to match the mass of rLcrV_S228_, which was unaffected by DTT treatment (Fig. 1B). DTT treatment of the *Y. pestis* LcrV_S228_ tryptic digest resulted in disappearance of the 1,985.79-Da signal, with appearance of a peptide with molecular mass of 1,680.71 Da, which on the basis of high-performance liquid chromatography (HPLC) retention time and molecular mass was indistinguishable from the corresponding rLcrV_S228_ peptide (Fig. 1B). On the basis of mass concordance, these data suggest that Cys^273^ of *Y. pestis* LcrV_S228_ forms a disulfide with glutathione (calculated residue monoisotopic mass of 305.07 Da). Confirming this conclusion, *Y. pestis* LcrV_S228_, but not *E. coli* rLcrV_S228_, was recognized by an antibody specific for glutathione (Fig. 1C).

10.1128/mBio.00646-17.1 FIG S1  Mass determination and Edman sequencing of LcrV_S228_. (A and B) Molecular mass spectra of (A) rLcrV_S228_ purified from cell extracts of *E. coli* DH5α(pKG48) or (B) LcrV_S228_ purified from culture supernatants of *Y. pestis* KLD29 Δ*lcrV*(pKG48) were reconstructed from the multiply charged ions recorded during combined liquid chromatography-electrospray ionization mass spectrometry (LC-ESI-MS). (A) The representative molecular mass spectrum of *E. coli* rLcrV_S228_ reveals a predominate isoform with a measured mass of 38,284.9 ± 1.4 Da, which corresponds to the full-length LcrV_S228_ protein, and a second isoform with measured mass of 38,152.8 ± 1.5 Da, which corresponds to the LcrV_S228_ protein lacking the initiator methionine residue. Data are means ± SEM (*n =* 9). (B) A representative molecular mass spectrum of *Y. pestis* LcrV_S228_ reveals a single predominant isoform with a measured mass of 38,594.4 ± 2.3 Da, which is 314.2 Da heavier than the calculated mass for full-length LcrV_S228_. Data are means ± SEM (*n =* 6). (C) LcrV_S228_ was purified from *Y. pestis* supernatants and subjected to Edman sequencing; the preponderant N-terminal amino acid released for each cycle (and its picomole abundance) is listed. Download FIG S1, TIF file, 5.1 MB. Copyright © 2017 Mitchell et al. 2017 Mitchell et al. This content is distributed under the terms of the Creative Commons Attribution 4.0 International license.

10.1128/mBio.00646-17.6 TABLE S1  Summary of mass spectrometry analysis of tryptic peptides from *Y. pestis* LcrV_S228_ and *E. coli* rLcrV_S228_. Download TABLE S1, DOCX file, 0.1 MB. Copyright © 2017 Mitchell et al. 2017 Mitchell et al. This content is distributed under the terms of the Creative Commons Attribution 4.0 International license.

10.1128/mBio.00646-17.7 TABLE S2  Tandem mass spectrometry of the 1,985.79-Da LcrV_S228_ peptide. Download TABLE S2, DOCX file, 0.1 MB. Copyright © 2017 Mitchell et al. 2017 Mitchell et al. This content is distributed under the terms of the Creative Commons Attribution 4.0 International license.

### *lcrV*_*C273A*_ accelerates *Y. pestis* killing of macrophages and increases type III injection of effectors into neutrophils.

**FIG 2:**
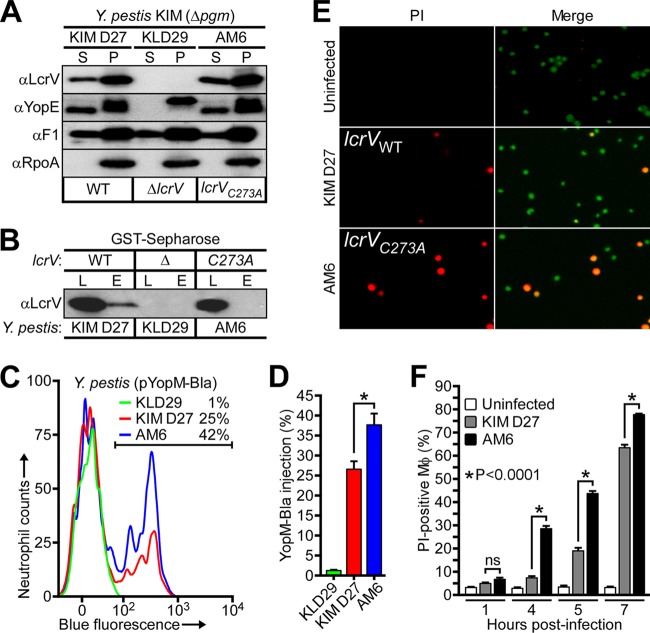
The *lcrV*_*C273A*_ mutation abolishes LcrV glutathionylation and accelerates *Y. pestis*-mediated macrophage death. (A) *Y. pestis* KIM D27 *lcrV*, KLD29 Δ*lcrV*, and AM6 *lcrV*_*C273A*_ were induced for type III secretion by growth at 37°C in M9-Casamino Acids (M9-Ca) minimal medium lacking exogenous calcium ions. Proteins in the supernatant (S) and bacterial pellet (P) were separated by centrifugation and identified by immunoblotting with rabbit antibodies specific for type III secretion substrates (LcrV and YopE), the secreted F1 pilus subunit (F1), and, as a fractionation control, cytoplasmic RNA polymerase subunit A (RpoA). (B) LcrV secreted by the indicated *Y. pestis* strains was assayed for glutathionylation by subjecting the culture supernatant to GST-Sepharose affinity chromatography. The load (L) and eluate (E) fractions were analyzed by immunoblotting with anti-LcrV. (C and D) Type III effector (YopM-Bla)-mediated cleavage of CCF2-AM-stained human polymorphonuclear leukocytes infected with *Y. pestis* KIM D27(pYopM-Bla), KLD29(pYopM-Bla), or AM6(pYopM-Bla) was analyzed via flow cytometry for blue fluorescence (YopM-Bla cleavage of CCF2-AM). (C) Representative histograms show the blue fluorescence traces of *Y. pestis*-infected neutrophils; the percentage of YopM-Bla-injected neutrophils (blue cells) is indicated above the gating scheme that was used to measure blue fluorescence above background. (D) Percentage of quantification of YopM-Bla-injected human neutrophils. Data are means ± standard errors of the means (SEM) (*n =* 3). *, *P* < 0.05, by two-tailed unpaired Student’s *t* test. (E and F) The kinetics of host cell death were examined by infecting murine J774.A1 macrophages with *Y. pestis* KIM D27 or AM6 and enumerating propidium iodide (PI)-positive cells. (E) Macrophages, either left uninfected or infected with *Y. pestis* KIM D27 or AM6, were stained with membrane-permeant (Hoechst [blue]) and membrane-impermeant (PI [red]) dyes and analyzed by fluorescence microscopy to determine *Y. pestis*-mediated macrophage death (PI positive, red, and Hoechst positive, magenta). (F) The kinetics of cell death were examined by quantifying PI-positive macrophages at timed intervals following *Y. pestis* infection. Data are means ± SEM (*n =* 3). *, *P* < 0.0001, and ns, not significant, by two-tailed unpaired Student’s *t* test.

Unlike wild-type (WT) *Y. pestis* (KIM D27), the Δ*lcrV* mutant (KLD29) did not secrete YopE via the type III pathway (Fig. 2A). The *lcrV*_*C273A*_ mutation did not impact type III secretion of YopE or LcrV in either low-calcium-induced broth cultures or during *Yersinia* infection of HeLa tissue cell cultures (Fig. 2A; see Fig. S2A and B in the supplemental material). We wondered whether *Y. pestis* KIM D27 Δ*pgm*, a nonpigmented plague strain (14) and parent of the *Y. pestis* KLD29 Δ*lcrV* variant (13), secretes glutathionylated LcrV. Because the glutathione-specific antibody, due to its low affinity (15), failed to detect LcrV in *Y. pestis* extracts, we used glutathione *S*-transferase (GST)-Sepharose as bait for enrichment of glutathionylated LcrV. Culture supernatants of *Y. pestis* KIM D27 and AM6 *lcrV*_*C273A*_ were subjected to GST-Sepharose affinity chromatography, and the eluate was analyzed with LcrV-specific antibody to reveal that wild-type LcrV, but not LcrV_C273A_, was selectively retained (Fig. 2B). Thus, *Y. pestis* KIM D27 LcrV is glutathionylated similarly to LcrV_S228_.

10.1128/mBio.00646-17.2 FIG S2  The codon substitution Cys^273^Ala, which precludes LcrV glutathionylation, does not affect *Y. pestis* type III secretion of Yop effectors. (A) Densitometric quantification of LcrV and YopE secreted by *Y. pestis* KIM D27 (Δ*pgm lcrV*), KLD29 (Δ*pgm* Δ*lcrV*), and AM6 (Δ*pgm lcrV*_*C273A*_) following growth at 37°C in M9-Ca minimal medium in the absence of exogenous calcium ions. The immunoreactive signal of each secreted protein (S) was normalized to its total abundance in the medium and pellet (S + P); the ratio of secreted protein to total protein, (S)/(S + P) is presented as a percent average. ND, no immunoreactive signal detected. (B) Compared to uninfected HeLa cells, infection by *Y. pestis* KIM D27 or AM6 causes actin cable rearrangements and cytotoxicity (cell rounding) as detected by differential interference contrast (DIC) or fluorescence microscopy of rhodamine-phalloidin-stained cells. (C and D) *Y. pestis* CO92 *pgm*^+^ *lcrV*, LQ1 *pgm*^+^ Δ*lcrV*, and TD1 *pgm*^+^ *lcrV*_*C273A*_ were induced for type III secretion by growth at 37°C in M9-Ca minimal medium with chelated calcium ions; secreted proteins (S) were separated from intact bacteria (P) by centrifugation. (C) Representative immunoblot analysis was performed by probing fractions with antibodies specific for type III secretion substrates (LcrV and YopE), the secreted F1 pilus subunit (F1), and a cytoplasmic fractionation control (RpoA). (D) The percentage of secretion of LcrV and YopE was determined by densitometry. ND, no immunoreactive signal detected. (E) LcrV secreted by the indicated *Y. pestis* strains was assayed for glutathionylation by subjecting the culture supernatant to GST-Sepharose affinity chromatography. The load (L) and eluate (E) fractions were analyzed by immunoblotting with anti-LcrV. All data are means ± SEM (*n =* 3). ns, not significant by two-tailed unpaired Student’s *t* test. Download FIG S2, TIF file, 22.3 MB. Copyright © 2017 Mitchell et al. 2017 Mitchell et al. This content is distributed under the terms of the Creative Commons Attribution 4.0 International license.

We investigated the impact of LcrV glutathionylation on *Y. pestis* infection of host immune cells. Following infection of human neutrophils, *Y. pestis* effector injection was measured by the conversion of the CCF2-AM fluorophore (green) to its YopM-Bla-cleaved chromophore (blue) (8), which occurred to a greater extent with *Y. pestis* AM6 relative to *Y. pestis* KIM D27 (Fig. 2C and D). During infection of murine J774.A1 macrophages, *Y. pestis* effector secretion triggers apoptotic cell death as well as a low level of pyroptotic cell death, and overall cytotoxicity can be quantified by enumerating propidium iodide (PI)-positive cells (16). *Y. pestis* AM6-mediated killing of J774.A1 macrophages occurred with significantly increased kinetics compared to *Y. pestis* KIM D27 (Fig. 2E and F). Prior work has demonstrated that, during *in vivo* pathogenesis as well as *ex vivo* infection of splenocytes, *Y. pestis* selectively targets neutrophils, macrophages, and dendritic cells for injection of Yops in order to evade and eliminate the innate immune responses of its mammalian host (8, 17). For this reason, our observation that glutathionylated LcrV impedes *Y. pestis* type III injection of Yop effectors and the killing of innate immune cells suggests that this modification could play a role in mediating *Y. pestis* target cell preference during bacterial pathogenesis.

### Glutathionylated LcrV is associated with enhanced virulence in rodent models of bubonic plague.

To examine whether the glutathionylation of LcrV impacts bubonic plague pathogenesis, we introduced the *lcrV*_*C273A*_ mutation into *Y. pestis* CO92 *pgm*^+^ *lcrV*, a pigmented plague strain isolated from a fatal case of human plague in Colorado (18), resulting in the generation of *Y. pestis* TD1 *pgm*^+^ *lcrV*_*C273A*_. Genome sequencing confirmed that apart from the Cys^273^Ala codon substitution in *lcrV*, *Y. pestis* CO92 and TD1 were genetically identical. *Y. pestis* CO92 and TD1 catalyzed type III secretion of LcrV and YopE at similar rates (Fig. S2C and D). GST-Sepharose affinity chromatography detected the glutathionylation of LcrV secreted by *Y. pestis* CO92 but not by the *lcrV*_*C273A*_ variant *Y. pestis* TD1 (Fig. S2E).

**FIG 3:**
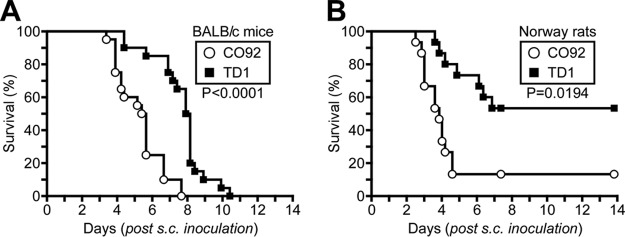
Glutathionylation of LcrV enhances bubonic plague pathogenesis. (A) Survival of cohorts of BALB/c mice (*n =* 20) infected via subcutaneous (s.c.) inoculation with 20 CFU of *Y. pestis* CO92 *lcrV* or *Y. pestis* TD1 *lcrV*_*C273A*_. WT versus *lcrV*_*C273A*_, *P* < 0.0001. (B) Survival of cohorts of Brown Norway rats (*n =* 15) infected via subcutaneous inoculation with 500 CFU of *Y. pestis* CO92 or TD1. WT versus *lcrV*_*C273A*_, *P* = 0.0194. Statistical analysis was performed using the Gehan-Breslow-Wilcoxon test. Data are representative of two independent experiments.

Nonenzootic mammalian hosts are highly susceptible to *Y. pestis* infection, a disease process that can be modeled by subcutaneous challenge of laboratory mice (cohorts of 6- to 8-week-old female BALB/c mice [*n =* 20]). Whereas the Δ*lcrV* mutant is avirulent in this model, *Y. pestis* CO92 causes lethal bubonic plague at a 50% lethal dose (LD_50_) of 1 to 5 bacteria (19). When analyzed in BALB/c mice by subcutaneous inoculation of 20 CFU *Y. pestis* CO92 or TD1, all mice succumbed to lethal plague disease by 11 days postchallenge (Fig. 3A). Nevertheless, mice infected by *Y. pestis* TD1 were slow to exhibit the characteristic features of plague disease (ruffled fur and hunched posture), and the median time to death of *Y. pestis* TD1-infected mice was delayed by 2.5 days relative to that of mice infected with *Y. pestis* CO92 (Fig. 3A). Animals were euthanized after exhibiting the symptoms of terminal plague disease: laterally recumbent and lethargic with rapid respiration. We examined *Y. pestis* colonization and dissemination in moribund BALB/c mice that were euthanized between 4 and 7 days postinfection. At the terminal stage of disease, the bacterial loads in the regional lymph node and spleen were similar for all *Y. pestis*-infected mice (see Fig. S3A in the supplemental material).

10.1128/mBio.00646-17.3 FIG S3  Kinetics of disease progression and host adaptive immune responses in bubonic plague-infected rodents. (A) BALB/c mice or (B to F) Brown Norway rats were infected by subcutaneous injection into the left inguinal fold with 20 CFU or 500 CFU, respectively, of *Y. pestis* CO92 *pgm*^+^ *lcrV* or *Y. pestis* TD1 *pgm*^+^ *lcrV*_*C273A*_. (A) Bacterial load in the regional lymph node and spleen of moribund BALB/c mice (*n =* 5) that were euthanized between 4 and 7 days postchallenge after exhibiting the symptoms of terminal illness. (B) Cohorts of Brown Norway rats (*n =* 5) were euthanized 72 h postchallenge to analyze the kinetics of bacterial replication in the regional lymph node as well as the kinetics of bacterial dissemination to the spleen. (C) Moribund rats (*n =* 5) that were euthanized between 4 and 7 days postchallenge were analyzed for disease progression by assessing the bacterial burden in the regional lymph node and spleen. (D to F) Bubonic plague-infected Brown Norway rats that survived for 14 days postchallenge were euthanized and necropsied. (D) Bacterial loads in the regional lymph node and spleen. (Note that since only two rats survived *Y. pestis* CO92 infection, necropsies were performed on two survivors—selected at random—of *Y. pestis* TD1 infection.) For rats surviving *Y. pestis* infection, serum IgG antibody titers for (E) LcrV or (F) capsular fraction F1 were quantified by ELISA. In panels A to D, the dotted lines represent the limit of detection, and red lines indicate geometric means. Statistical analysis was performed using the Mann-Whitney *U* test. In panels E and F, red lines indicate arithmetic means. Statistical analysis was performed using the two-tailed unpaired Student’s *t* test. All data are representative of two independent experiments. Download FIG S3, TIF file, 27.9 MB. Copyright © 2017 Mitchell et al. 2017 Mitchell et al. This content is distributed under the terms of the Creative Commons Attribution 4.0 International license.

Bubonic plague pathogenesis was also examined in Brown Norway rats (*Rattus norvegicus*), an enzootic species that is moderately resistant to challenge with *Y. pestis* CO92, which causes bubonic disease in rats at an LD_50_ of 1,000 to 5,000 CFU (20). Cohorts of 6-week-old female *Rattus norvegicus* animals (*n =* 15) were infected by subcutaneous injection into the left inguinal fold with 500 CFU *Y. pestis* CO92 or TD1. Whereas only 13% (2/15) of the rats survived bubonic plague infection with *Y. pestis* CO92, 53% (8/15) of infected rats survived the *Y. pestis* TD1 challenge (Fig. 3B). Bubonic plague-infected rats (*n =* 5) were also euthanized at 3 days postchallenge to monitor disease progression. Compared to *Y. pestis* CO92-infected rats, rats infected with *Y. pestis* TD1 displayed a reduction in the kinetics of replication in the regional lymph node (4.4 × 10^7^ CFU CO92 versus 1.5 × 10^5^ CFU TD1; *P* = 0.24) as well as in the kinetics of dissemination to the spleen (3.2 × 10^8^ CFU CO92 versus 1.4 × 10^6^ CFU TD1; *P* = 0.75); due to the small cohort size of rats surviving *Y. pestis* CO92 challenge, the observed differences were not statistically significant (Fig. S3B).

Rats euthanized between 4 and 7 days postchallenge were analyzed for disease progression. At the terminal stage of bubonic plague disease, *Y. pestis* CO92 and TD1 had replicated to similar levels in the regional lymph node as well as the spleen (Fig. S3C). Rats that survived bubonic plague infection for 14 days were euthanized and necropsied. All survivors of *Y. pestis* CO92 and TD1 challenge lacked detectable levels of plague bacteria in their spleens, nor was there a statistically significant difference in the number of viable plague bacteria in the regional lymph nodes of surviving animals (1.2 × 10^7^ CFU CO92 versus 1.0 × 10^2^ CFU TD1; *P* = 0.67) (Fig. S3D). Moreover, no difference in antibody titers against LcrV or capsular fraction 1 (F1), the predominant plague antigen, was detectable in serum from *Y. pestis* CO92- and TD1-infected animals (Fig. S3E and F). Together these data indicate that a single amino acid substitution (Cys^273^Ala) associated with loss of LcrV glutathionylation increases the survival of bubonic plague-infected rats.

### Serine substitution at LcrV residue 273.

The plague pathogen evolved in the rodent population of China (21), where isolates are still scattered over multiple phylogenetic branches (22, 23). The pCD1-encoded type III secretion machine and its *lcrV* gene are conserved among all members of this clade, albeit that a polymorphism substituting LcrV Cys^273^Ser is found in some isolates of the *Y. pestis* biovar *microtus*, which does not cause plague in humans (24), and in the *Y. pestis* biovar *pestoides* Angola isolate (25) (see Fig. S4A in the supplemental material). A previous study reported that the *lcrV*_*C273S*_ polymorphism impacts the oligomerization of LcrV and represents one out of four highly variable regions of LcrV that are unique to nonepidemic strains of *Y. pestis* (26). Since the *lcrV*_*C273A*_ mutation was associated with attenuated virulence in animal models of bubonic plague, we asked whether the *lcrV*_*C273S*_ mutation causes a similar phenotype when introduced into modern plague strains. The codon substitution Cys^273^Ser was introduced into the *lcrV* alleles of *Y. pestis* KIM D27 and *Y. pestis* CO92 to generate *Y. pestis* AM15 Δ*pgm lcrV*_*C273S*_ and *Y. pestis* DE1 *pgm*^+^ *lcrV*_*C273S*_, respectively. *Y. pestis* KIM D27 *lcrV* and AM15 *lcrV*_*C273S*_ catalyzed type III secretion of YopE and LcrV into the extracellular medium at similar rates (Fig. S4B and C). LcrV secreted by *Y. pestis* AM15 was not retained during GST-Sepharose affinity chromatography, suggesting that Ser^273^ was not modified with glutathione (Fig. S4D). To validate this conjecture, LcrV_S228 C273S_ was affinity purified from the culture supernatant of *Y. pestis* KLD29 Δ*lcrV*(pAM199) (Fig. S4E). When analyzed by matrix-assisted laser desorption ionization–time of flight mass spectrometry (MALDI-TOF MS) along with recombinant rLcrV_S228 C273S_ purified from *E. coli* DH5α(pAM199) lysates, we observed *m*/*z* 38,261.34 for LcrV_S228 C273S_ and *m*/*z* 38,286.09 for rLcrV_S228 C273S_ (Fig. S4E; see Table S4 in the supplemental material). These measurements approximate the calculated mass for unmodified LcrV_S228 C273S_ (38,264.16 Da), suggesting that LcrV_S228 C273S_ is not posttranslationally modified with glutathione (Table S4). Following infection of human neutrophils, *Y. pestis* AM15(pYopM-Bla), compared to *Y. pestis* KIM D27(pYopM-Bla), caused a small but statistically insignificant increase in the conversion of CCF2-AM fluorophore to the YopM-Bla-cleaved chromophore (blue) (Fig. S4F and G). *Y. pestis* CO92 *lcrV* and *Y. pestis* DE1 *lcrV*_*C273S*_ exported type III effectors into the extracellular milieu at similar levels (Fig. S4H and I). When analyzed in a mouse model of bubonic plague following subcutaneous inoculation of 20 CFU, all *Y. pestis* CO92- or DE1-infected animals succumbed to lethal plague disease. Similar to mice infected with *Y. pestis* TD1 *lcrV*_*C273A*_, *Y. pestis* DE1 *lcrV*_*C273S*_ infection caused a delay in time to death (Fig. S4J). Taken together, these data suggest that the codon 273 polymorphism (*lcrV*_*C273S*_) observed in *Y. pestis* biovar *pestoides* and *microtus* isolates (26) prevents LcrV glutathionylation and affects bubonic plague pathogenesis.

10.1128/mBio.00646-17.4 FIG S4  The codon substitution Cys^273^Ser abolishes *Y. pestis* posttranslational modification of LcrV and attenuates virulence in a mouse model of bubonic plague. (A) *Y. pestis* isolates and nucleotide sequences at codons 272 to 273 (nucleotides 814 to 819 of *Y. pestis* CO92 *lcrV*) that impact codon 273 (Cys or Ser). (B and C) Following growth at 37°C in M9-Ca minimal medium lacking calcium ions, cultures of *Y. pestis* KIM D27 Δ*pgm lcrV* or AM15 Δ*pgm lcrV*_*C273S*_ were fractionated and analyzed for type III secretion. (B) Representative immunoblot analysis was performed by probing supernatant (S) and pellet (P) fractions with the indicated rabbit antibodies. (C) Percentages of secretion of YopE and LcrV were determined by densitometric quantification of immunoreactive signals. (D) *Y. pestis* culture supernatants were subjected to GST-Sepharose affinity chromatography. The load (L) and eluate (E) fractions were immunoblotted with LcrV-specific antisera to assay for the secretion of glutathionylated LcrV. (E) *Y. pestis* KLD29 Δ*lcrV* and *E. coli* DH5α strains expressing LcrV_S228 C273S_ (pAM199) were propagated in LB broth at 37°C. LcrV was affinity purified from *Y. pestis* culture supernatants (LcrV_S228 C273S_) or *E. coli* lysates (rLcrV_S228 C273S_), visualized by Coomassie-stained SDS-PAGE, and analyzed by MALDI-TOF MS. The experimentally observed *m/z* of each LcrV_S228 C273S_ preparation is indicated. (F and G) Following infection with *Y. pestis* KIM D27(pYopM-Bla), KLD29(pYopM-Bla), or AM15(pYopM-Bla), human neutrophils were stained with CCF2-AM and analyzed by flow cytometry for blue fluorescence indicative of type III effector (YopM-Bla)-mediated cleavage of CCF2-AM. (F) Representative histograms show the blue fluorescence traces of *Y. pestis*-infected neutrophils; the percentage of YopM-Bla-injected neutrophils (blue cells) is indicated above the gating scheme that was used to measure blue fluorescence above background. (G) Percentage of quantification of YopM-Bla-injected human neutrophils. (H and I) *Y. pestis* CO92 *pgm*^+^ *lcrV* and DE1 *pgm*^+^ *lcrV*_*C273S*_ were evaluated for type III secretion following growth at 37°C in M9-Ca minimal medium in the absence of exogenous calcium ions. (H) Representative immunoblot analysis of supernatant (S) and pellet (P) fractions. (I) Percentages of secretion of LcrV and YopE were determined by densitometry. (J) Survival of cohorts of BALB/c mice (*n =* 15) infected by subcutaneous injection into the left inguinal fold with 20 CFU of *Y. pestis* CO92 or DE1. The figure presents pooled data that were gathered from two independent experiments. For WT versus *lcrV*_*C273S*_, *P* = 0.0351 by Gehan-Breslow-Wilcoxon test. In panels C, G, and I, data are means ± SEM (*n =* 3). ns, not significant by two-tailed unpaired Student’s *t* test. Download FIG S4, TIF file, 25.8 MB. Copyright © 2017 Mitchell et al. 2017 Mitchell et al. This content is distributed under the terms of the Creative Commons Attribution 4.0 International license.

### Decreased LcrV-RPS3 association is correlated with increased macrophage apoptosis and diminished cytokine release.

**FIG 4:**
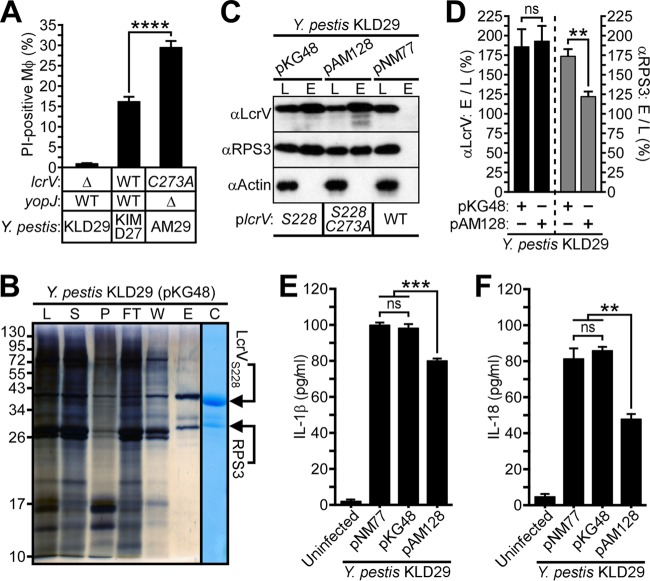
LcrV binds to macrophage RPS3 and modulates host inflammatory responses. (A) *Y. pestis*-mediated death of J774.A1 macrophages was assessed 3 h postinfection by propidium iodide staining. (B) *Y. pestis* KLD29 *lcrV*_*S228*_(pKG48)-infected J774.A1 macrophages were subjected to Strep-Tactin affinity chromatography. The crude lysate (L), lysate supernatant (S), lysate pellet (P), flowthrough (FT), wash (W), and eluate (E) fractions were collected and analyzed by silver- and Coomassie-stained SDS-PAGE; bottom-up proteomics was used to identify the indicated protein bands as LcrV_S228_ and macrophage ribosomal protein S3 (RPS3). (C and D) Immunoblotting of lysate (L) and eluate (E) fractions generated during Strep-Tactin affinity chromatography reveals variable association between LcrV variants and RPS3 in J774.A1 macrophages infected with *Y. pestis* KLD29 Δ*lcrV* expressing wild-type *lcrV* (pNM77), *lcrV*_*S228*_ (pKG48), or *lcrV*_*S228 C273A*_ (pAM128). (C) Representative immunoblot analysis of LcrV and RPS3 purified from *Y. pestis*-infected macrophages; actin levels were used as a loading control. (D) Densitometric quantification of the interaction between LcrV_S228_ and RPS3. Immunoreactive signals of Strep-Tactin-purified LcrV and RPS3 were normalized to the corresponding actin band; the ratio of eluate to lysate (E to L) is presented as a percent average. (E and F) Supernatants from J774.A1 macrophages, either left uninfected or infected with the indicated *Y. pestis* strains, were assayed by ELISA for (E) IL-1β or (F) IL-18. All data are means ± SEM (*n =* 3). **, *P* < 0.01; ***, *P* < 0.001; ****, *P* < 0.0001; ns, not significant.

Earlier work demonstrated that YopJ, a type III effector injected into host immune cells, functions as an acetyltransferase that inhibits NF-κB and mitogen-activated protein kinase (MAPK) signaling, triggers caspase-1 activation and interleukin-1β (IL-1β) and IL-18 secretion, and also promotes target cell death in *Y. pestis*-infected macrophages (27, 28). Furthermore, *yopJ* has been shown to be dispensable for bubonic plague pathogenesis in *Y. pestis*-infected Brown Norway rats (29). Since loss of LcrV glutathionylation attenuated plague pathogenesis, we asked whether *yopJ* was required for or contributed to increased cytotoxicity *in vitro* by generating an in-frame *yopJ* deletion strain by allelic exchange with *Y. pestis* AM6. Significantly, the *lcrV*_*C273A*_-mediated increase in macrophage cell death during *Y. pestis* infection did not require *yopJ*, as *Y. pestis* AM29 *lcrV*_*C273A*_ Δ*yopJ* also promoted increased macrophage cytotoxicity compared with *Y. pestis* KIM D27 (Fig. 4A; see Fig. S5A in the supplemental material).

10.1128/mBio.00646-17.5 FIG S5  *yopJ* is dispensable for *lcrV*_*C273A*_-mediated killing of *Y. pestis*-infected macrophages, and type III secretion is not impacted by loss of glutathione synethase (*gshB*). (A) An in-frame deletion of *yopJ* was introduced by allelic exchange with wild-type *Y. pestis* KIM D27 and its *lcrV*_*C273A*_ variant, *Y. pestis* AM6, to generate *Y. pestis* AM27 *lcrV* Δ*yopJ* and *Y. pestis* AM29 *lcrV*_*C273A*_ Δ*yopJ*, respectively. Murine J774.A1 macrophages were infected for 3 h with the indicated *Y. pestis* strains, and macrophage cell death was quantified by enumeratng propidium iodide (PI)-positive cells. (B) Partial HPLC chromatograms include a reaction blank that was used to identify peaks originating from the derivatization reaction in the absence of sample-derived thiols, a standard mixture containing 10 pmol of Cys, GSH, and γ-EC that was used to assign peak positions for the corresponding thiol-mBBr adducts on the basis of retention time, and sample runs for TMH medium and LB broth. (C and D) Densitometric quantification of LcrV_S228_ and YopE secreted by *Y. pestis* KLD29 Δ*lcrV*(pKG48) and AM43 Δ*lcrV* Δ*gshB*(pKG48) following growth in the presence (+Ca^2+^) or absence (−Ca^2+^) of calcium ions at 37°C in (C) LB broth or (D) TMH medium. The immunoreactive signal of each secreted protein (S) was normalized to its total abundance in the medium and pellet (S + P); the ratio of secreted protein to total protein, (S)/(S + P), is presented as a percent average. All data are means ± SEM (*n =* 3). ****, *P* < 0.0001, and ns, not significant, by two-tailed unpaired Student’s *t* test. Download FIG S5, TIF file, 30.9 MB. Copyright © 2017 Mitchell et al. 2017 Mitchell et al. This content is distributed under the terms of the Creative Commons Attribution 4.0 International license.

To investigate the molecular mechanism for LcrV-mediated killing of immune cells, we purified LcrV_S228_ via affinity chromatography from *Y. pestis*-infected J774.A1 macrophages and used mass spectrometry to identify proteins separated by Coomassie-stained SDS-PAGE (Fig. 4B; see Table S3 in the supplemental material). Infection of macrophages with *Y. pestis* KLD29 expressing LcrV_S228_ (pKG48), but not with *Y. pestis* KLD29 expressing wild-type LcrV (pNM77), led to purification of LcrV_S228_ and ribosomal protein S3 (RPS3), a component of the eukaryotic 40S ribosomal subunit and a regulator of DNA repair, apoptosis, and innate immune responses (30) (Fig. 4B and C; Table S3). Strikingly, analysis of J774.A1 macrophages infected with *Y. pestis* KLD29 expressing LcrV_S228 C273A_ (pAM128) revealed that copurification of RPS3 with LcrV_S228 C273A_ was diminished (Fig. 4C and D). Earlier work has shown that pathogenic *E. coli* effectors, injected into immune cells by type III secretion machines, target RPS3 to inhibit host immune defenses by modulating RPS3/NF-κB-mediated signaling and proinflammatory responses (30–32). We wondered whether the differential association between RPS3 and LcrV variants that was observed for *Y. pestis*-infected macrophages influences host inflammatory responses. The impact of LcrV glutathionylation on host inflammatory responses in *Y. pestis*-infected J774.A1 macrophages undergoing apoptotic or pyroptotic cell death was assessed by quantifying the caspase 1/11-induced cytokines IL-1β and IL-18 (33). Compared to *Y. pestis* KLD29 expressing wild-type LcrV (pNM77) or LcrV_S228_ (pKG48), *Y. pestis* KLD29 expressing LcrV_S228 C273A_ (pAM128) caused a significant increase in nonpyroptotic cell death of J774.A1 macrophages, as suggested by reduced levels of IL-1β and IL-18 cytokine release (Fig. 4E and F). Thus, the association of LcrV with RPS3 may be involved in regulating macrophage cell death during *Y. pestis* infection and, reciprocally, perturbation of the RPS3 association with LcrV in *Y. pestis*-infected macrophages may be responsible for the decrease in disease severity as well as the increase in apoptotic cell death observed for the *lcrV*_*C273A*_ variants (Fig. 2F, 3A and B, and 4D).

10.1128/mBio.00646-17.8 TABLE S3  Peptide mass fingerprinting by LC-MS/MS identifies macrophage RPS3 as a ligand of translocated LcrV. Download TABLE S3, DOCX file, 0.1 MB. Copyright © 2017 Mitchell et al. 2017 Mitchell et al. This content is distributed under the terms of the Creative Commons Attribution 4.0 International license.

### LcrV is modified via disulfide formation with extracellular glutathione.

**FIG 5:**
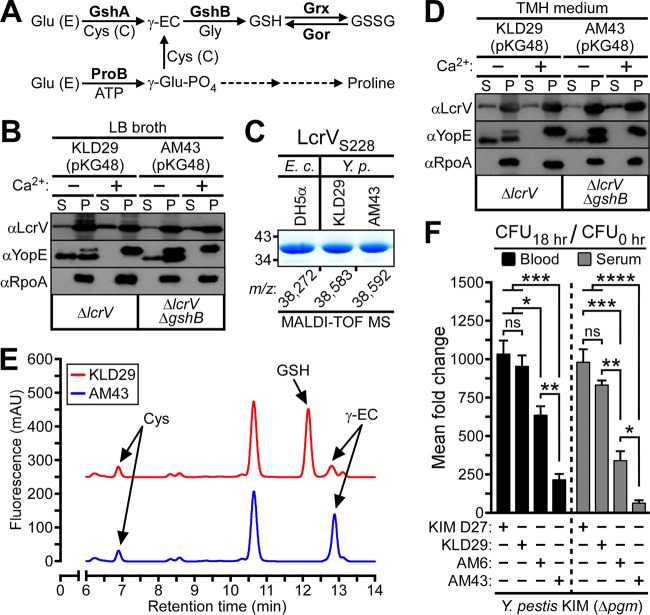
Extracellular glutathione modifies secreted LcrV and promotes *Y. pestis* survival in blood. (A) Pathways of glutathione biosynthesis in *E. coli*. Glu (E), glutamic acid; Cys (C), cysteine; GshA, γ-glutamylcysteine synthetase; GshB, glutathione synthetase; ProB, γ-glutamyl kinase; γ-EC, γ-glutamylcysteine; GSH, glutathione; GSSG, glutathione disulfide; Gor, glutathione reductase; Grx, glutaredoxin; γ-glutamyl-PO_4_, γ-glutamyl phosphate. (B) *Y. pestis* KLD29 Δ*lcrV lcrV*_*S228*_(pKG48) and AM43 Δ*lcrV* Δ*gshB lcrV*_*S228*_(pKG48) were grown at 37°C in LB broth, in either the presence or absence of calcium ions (Ca^2+^), and assayed for type III secretion by immunoblotting; proteins secreted into the supernatant (S) were separated from intact bacteria (P) by centrifugation. (C) *Y. pestis* and *E. coli* strains expressing LcrV_S228_ (pKG48) were propagated in LB broth. LcrV was purified from *Y. pestis* supernatants (LcrV_S228_) or *E. coli* extracts (rLcrV_S228_), visualized by Coomassie-stained SDS-PAGE, and analyzed by MALDI-TOF MS to reveal the *m/z* of each LcrV_S228_ purification sample. (D) *Y. pestis* strains were analyzed for calcium-regulated type III secretion in TMH medium. (E) Partial HPLC chromatograms of LMW thiol-mBBr derivatives extracted from *Y. pestis* cultures grown to the stationary phase in TMH medium. Peaks corresponding to Cys, GSH, and γ-EC were assigned on the basis of retention time by comparison to a chromatogram of thiol standards. (F) Approximately 10^5^ CFU of *Y. pestis* KIM D27 *lcrV*, KLD29 Δ*lcrV*, AM6 *lcrV*_*C273A*_, or AM43 Δ*lcrV* Δ*gshB* was inoculated into 4 ml of defibrinated sheep blood or heat-inactivated sheep serum. Culture aliquots were removed before and after 18 h of growth at 37°C and plated on LB agar to enumerate bacterial load. *Y. pestis* growth was calculated as the mean fold increase in bacteria at the time of inoculation (CFU_0 h_) to bacteria recovered after the 37°C incubation (CFU_18 h_). Data are means ± SEM (*n =* 3). *, *P* < 0.05, **, *P* < 0.01, ***, *P* < 0.001, and ****, *P* < 0.0001, and ns, not significant, by one-way ANOVA with Tukey’s multiple-comparison posttest.

In *E. coli*, a Gram-negative enterobacterium and relative of *Y. pestis*, the tripeptide glutathione (γ-glutamyl-cysteinyl-glycine [GSH]) can be synthesized through two pathways. The *de novo* GSH biosynthetic pathway is a two-step process catalyzed by the ATP-dependent enzymes γ-glutamylcysteine synthetase (GshA) and glutathione synthetase (GshB), whereby glutamate (Glu [E]) and cysteine (Cys [C]) are first ligated by GshA to form γ-glutamylcysteine (γ-EC), which is then conjugated to glycine by GshB to generate GSH (34, 35) (Fig. 5A). An alternate pathway for GSH synthesis was characterized in a Δ*gshA* mutant strain of *E. coli* and involves the first enzyme of the proline biosynthetic pathway, γ-glutamyl kinase (ProB), catalyzing the conversion of glutamate to γ-glutamyl phosphate, which in a condensation reaction with cysteine, generates γ-glutamylcysteine that is subsequently ligated to glycine by GshB to form GSH (36) (Fig. 5A). Glutathione is oxidized at its sulfhydryl to generate glutathione disulfide (GSSG), which, in turn, is reduced by the NADPH-dependent enzyme glutathione reductase (Gor) in order to maintain a high GSH/GSSG ratio in the bacterial cytoplasm (37) (Fig. 5A).

We asked whether LcrV glutathionylation occurs in the *Y. pestis* cytoplasm via thiol-disulfide exchange with GSSG or a mixed disulfide prior to its secretion and capping of the type III needle complex. As GshB is required for both the *de novo* and compensatory pathways of GSH synthesis, we generated an in-frame Δ*gshB* deletion strain by allelic exchange with *Y. pestis* KLD29. We hypothesized that Δ*gshB* mutant *Y. pestis* would be unable to synthesize glutathione and thereby preclude cytoplasmic, but not extracellular, glutathionylation of LcrV. When analyzed for type III secretion in Luria-Bertani (LB) broth, a rich medium that contains glutathione (Fig. S5B), *Y. pestis* KLD29 Δ*lcrV*(pKG48) and *Y. pestis* AM43 Δ*lcrV* Δ*gshB*(pKG48) exhibited calcium-regulated secretion of LcrV_S228_ and YopE (Fig. 5B; Fig. S5C). To assess glutathionylation of LcrV secreted by *Y. pestis* during growth in LB broth, LcrV_S228_ was affinity purified from *Y. pestis* KLD29(pKG48) and *Y. pestis* AM43(pKG48) and analyzed by MALDI-TOF MS to reveal *m/z* 38,583.10 and 38,592.16, respectively, which approximate the calculated mass for glutathionylated LcrV_S228_ (38,585.54 Da) (Fig. 5C; Table S4). Compared to the *m/z* of rLcrV_S228_ purified from *E. coli* DH5α(pKG48) grown in LB broth (38,271.66), these data suggest that LcrV_S228_ secreted by Δ*gshB* mutant *Y. pestis* is glutathionylated. Confirming this result, DTT treatment collapsed the measured masses of *Y. pestis* LcrV_S228_ to match the mass of rLcrV_S228_, which was unaffected by DTT treatment (Table S4). These data establish that LcrV_S228_ secreted by Δ*gshB* mutant *Y. pestis* during growth in LB broth is modified with glutathione.

10.1128/mBio.00646-17.9 TABLE S4  MALDI-TOF MS analysis of LcrV_S228_ and LcrV_S228 C273S_ purified from *Y. pestis* supernatants or *E. coli* extracts. Download TABLE S4, DOCX file, 0.1 MB. Copyright © 2017 Mitchell et al. 2017 Mitchell et al. This content is distributed under the terms of the Creative Commons Attribution 4.0 International license.

Following growth at 37°C in thoroughly modified Higuchi (TMH) medium, a chemically defined minimal medium that lacks glutathione (Fig. S5B), *Y. pestis* KLD29(pKG48) and *Y. pestis* AM43(pKG48) exhibited calcium-regulated export of LcrV_S228_ and YopE at similar rates (Fig. 5D; Fig. S5D). To investigate whether the Δ*gshB* mutation was sufficient to abolish bacterial glutathione synthesis, *Y. pestis* KLD29 and AM43 were propagated to stationary phase in TMH medium, and nonprotein thiols were acid extracted, reduced by treatment with tris(2-carboxyethyl)phosphine (TCEP), derivatized with the thiol-specific reagent monobromobimane (mBBr), and analyzed by high-performance liquid chromatography (HPLC) for the total abundance of cysteine, glutathione, and γ-glutamylcysteine in cell extracts. Compared to *Y. pestis* KLD29, the HPLC chromatogram of *Y. pestis* AM43 revealed an undetectable level of glutathione alongside a significant accumulation of its biosynthetic precursor, γ-glutamylcysteine (Fig. 5E). We infer from these data two conclusions. First, the glutathionylation of LcrV occurs after the protein is exported from the *Y. pestis* cytoplasm to cap the type III needle complex, and second, the molecular mechanism underpinning LcrV glutathionylation is thiol-disulfide exchange with oxidized glutathione in the extracellular milieu.

### Glutathione promotes *Y. pestis* growth in mammalian blood and heat-inactivated serum.

The survival of *Y. pestis* in the blood of its mammalian host—as occurs during its initial trafficking from the site of fleabite inoculation to the regional lymph node and, more significantly, during its systemic hematogenous dissemination to colonize distal host tissues—is essential for bubonic plague pathogenesis (38). Recent studies of pathogenic bacteria identified mutations that, either by abolishing *de novo* glutathione synthesis or abrogating bacterial import of host-derived glutathione, result in attenuated virulence in animal models of infection and/or impaired survival in vertebrate blood (2). Glutathione has also been shown to play a significant role in bacterial pathogenesis by functioning as a thiol redox buffer that enables blood-borne pathogens to rapidly acclimate to the high osmolality and oxidative stresses inherent to animal blood (6). For these reasons, we investigated whether the *lcrV*_*C273A*_ and Δ*gshB* mutations impact *Y. pestis* survival in defibrinated sheep blood by enumerating the mean fold change in viable CFU following 18 h of growth at 37°C. Unlike *Y. pestis* KIM D27 *lcrV* (1,034-fold increase in CFU), *Y. pestis* KLD29 Δ*lcrV* (954-fold increase), or *Y. pestis* AM6 *lcrV*_*C273A*_ (636-fold increase), the CFU of *Y. pestis* AM43 Δ*lcrV* Δ*gshB* increased only 216-fold (Fig. 5F). Animal blood contains immune cells and factors facilitating complement-mediated cell lysis. To determine whether these antimicrobial properties of blood account for the diminished replication of Δ*gshB* mutants, *Y. pestis* survival was assayed in heat-inactivated sheep serum. Compared to the increase in viable CFU observed for *Y. pestis* KIM D27 (980-fold), *Y. pestis* KLD29 (832-fold), and *Y. pestis* AM6 (342-fold) following 18 h of growth at 37°C, growth in heat-inactivated sheep serum was diminished for *Y. pestis* AM43 (64-fold increase in CFU) (Fig. 5F). This result suggests that neither immune cells nor the complement system of defibrinated sheep blood is responsible for the observed growth defects of *lcrV*_*C273A*_ or Δ*gshB* variant *Y. pestis* strains. As Δ*gshB* mutant *Y. pestis* exhibited a pronounced growth defect in both blood and heat-inactivated serum, we presume that the Δ*gshB* mutation may also attenuate the pathogenesis of *Y. pestis* in animal models of bubonic plague.

Although *Y. pestis* LcrV has been the subject of intensive study for decades, a complete understanding of its multifaceted contributions to plague pathogenesis continues to evolve. Here we demonstrate that LcrV is modified by host-derived glutathione following its export to the tip of the type III secretion machine. LcrV glutathionylation moderates the rate of *Y. pestis* type III effector injection into immune cells and enhances *Y. pestis* virulence in mouse and rat models of bubonic plague. We arrived at these conclusions when comparing the phenotypes of wild-type (Cys^273^) and *lcrV* mutant (Ala^273^) strains, where the amino acid side chains of LcrV residue 273 both display hydrophobic properties (methylene-sulfhydryl in cysteine and methyl in alanine); however, alanine, in contrast to cysteine, cannot be glutathionylated. On the other hand, the *lcrV* Ser^273^ mutant, in which the methylene-hydroxyl side chain at residue 273 exerts a polar character, displayed weaker phenotypes in both *Y. pestis* type III injection and plague virulence assays. We presume that the side chain of LcrV residue 273 occupies a critical position that may directly affect the type III injection of *Y. pestis* effectors. LcrV glutathionylation promotes also the association of LcrV with host RPS3 and suppresses the apoptotic killing of *Y. pestis*-infected macrophages. We think it is likely that the abundance of glutathione and the strong oxidizing environment in mammalian blood promote Cys^273^ glutathionylation. It is, however, not clear, whether LcrV glutathionylation is impacted during *Y. pestis* type III injection of effectors into immune cells, as the disulfide of LcrV glutathione may be exposed to the reducing environment of the host cell cytoplasm. LcrV glutathionylation promotes necroptotic cell death and IL-1β and IL-18 secretion, triggering inflammatory responses to plague infection that presumably enable *Y. pestis* to rapidly kill large numbers of immune cells via the type III mechanism or to disseminate with immune cells through host tissues. These activities of glutathionylated LcrV are correlated with its association with RPS3, a protein that resides in the cytoplasm of host cells. We presume that LcrV may gain access to RPS3 via the *Y. pestis* type III mechanism; however, the molecular details for these events are not known. In the absence of LcV glutathionylation, *Y. pestis* injects larger amounts of effectors into immune cells, an alteration that is associated with increased apoptotic cell death and diminished secretion of IL-1β and IL-18. This reduction in host inflammatory responses may delay *Y. pestis* type III-mediated killing of immune cells and the distribution of plague bacteria in host tissues, thereby attenuating *Y. pestis* virulence in animal models of plague pathogenesis. Intriguingly, the site of LcrV glutathionylation is located in a domain that is also known to modulate host immune responses by stimulating IL-10 release (39). To the best of our knowledge, LcrV glutathionylation represents the first report of a bacterial secreted virulence factor that is modified by glutathione. We wondered whether other virulence factors, whether secreted by *Y. pestis* or other bacterial pathogens, may likewise be modified by host-derived glutathione and contribute to the pathogenesis of infectious diseases. The *lcrV* genes of two related pathogens, *Yersinia pseudotuberculosis* and *Yersinia enterocolitica*, also carry a cysteine codon at position 273. In contrast, the genes for needle cap proteins in the type III pathways of many other bacterial pathogens are not endowed with cysteine codons. Thus, glutathionylation of the type III secretion needle cap protein may be unique to *Yersinia* spp. and not a universal attribute of pathogenic bacteria.

## MATERIALS AND METHODS

### Ethical conduct, biosafety, and biosecurity.

Experiments with blood from human volunteers were performed with protocols that had been reviewed, approved, and supervised by the University of Chicago’s Institutional Review Board (IRB). Animal research was performed in accordance with institutional guidelines following experimental protocol review, approval, and supervision by the Institutional Animal Care and Use Committee at The University of Chicago. Experiments with *Y. pestis* were performed in biosafety level 3 (BSL-3)/animal BSL3 (ABSL3) containment. The University of Chicago Select Agent Program is approved and routinely inspected by both Institutional Biosafety Committee and Centers for Disease Control and Prevention (CDC) officials. The manuscript was analyzed for dual-use research of concern (DURC): i.e., the description of mutations that may increase the virulence of natural isolates of *Y. pestis* or affect their ability to cause fulminant plague or epizootic outbreaks in rats or other host species. The analysis was reviewed by The University of Chicago Institutional Biosafety Committee DURC Task Force (DTF) and by the funding agency, the National Institute of Allergy and Infectious Diseases, which agreed with the investigators’ assessment that the findings presented in the manuscript do not present DURC.

### Bacterial strains and plasmids.

The strains, plasmids, and primers used in this study are listed in Table S5 in the supplemental material (8, 13, 16, 18, 19, 40–43). Overnight cultures of *Y. pestis* were propagated at 26°C in heart infusion broth (HIB), M9-Casamino Acids (M9-Ca) minimal medium, or thoroughly modified Higuchi’s (TMH) medium. *E. coli* strains were propagated at 37°C in Luria-Bertani (LB) broth. When necessary, the growth medium was supplemented with ampicillin (100 µg/ml), kanamycin (50 µg/ml), or chloramphenicol (20 µg/ml) for plasmid maintenance. The *lcrV*_*C273A*_ mutant strains (AM6 and TD1), the *lcrV*_*C273S*_ mutant strains (AM15 and DE1), and the in-frame deletions in *yopJ* and *gshB* were generated via allelic exchange in *Y. pestis* KIM D27 and *Y. pestis* CO92, as previously described (16). For the construction of plasmids pAM128 (*lcrV*_*S228 C273A*_) and pAM199 (*lcrV*_*S228 C273S*_), the QuikChange Lightning site-directed mutagenesis kit was used to introduce the Cys^273^ codon substitution into the *lcrV*_*S228*_ allele carried by pKG48. For genome sequencing, DNA was extracted from *Y. pestis* strains CO92, TD1 *lcrV*_*C273A*_, and DE1 *lcrV*_*C273S*_ using the Wizard Genomic DNA purification kit (Promega). The Next Generation Sequencing Core at Argonne National Laboratory used extracted DNA to generate TruSeq libraries (average insert size, 400 to 500 bp) that were sequenced on an Illumina MISeq system (250 by 250 bp, paired-end reads). The FASTQ formatted files of raw genomic sequencing data were analyzed with Geneious R9.0 to assemble and compare genomic sequences for single nucleotide polymorphism (SNP), indel, and structural variations. Compared with *Y. pestis* CO92, *Y. pestis* TD1 and *Y. pestis* DE1 carried the *lcrV*_*C273A*_ and *lcrV*_*C273S*_ mutations, respectively, without additional mutations affecting the coding sequence of proteins.

10.1128/mBio.00646-17.10 TABLE S5  Bacterial strains, plasmids, and primers used in this study. Download TABLE S5, DOCX file, 0.1 MB. Copyright © 2017 Mitchell et al. 2017 Mitchell et al. This content is distributed under the terms of the Creative Commons Attribution 4.0 International license.

### LcrV purification.

For purification of LcrVS_228_, Y. *pestis* KLD29(pKG48) overnight cultures were diluted 1:20 into 4 liters HIB, grown for 2 h at 26°C, supplemented with 5 mM CaCl_2_ and 1 mM isopropyl-β-d-thiogalactopyranoside (IPTG), and propagated at 37°C for 3 h. Bacteria were sedimented at 8,500 × *g* for 7 min. Supernatant was filtered through a 0.22-µm-pore membrane (Millipore). Proteins were precipitated with 60% ammonium sulfate and overnight incubation at 4°C. Protein precipitate was sedimented at 12,500 × *g* for 15 min, suspended in 16 ml of column buffer (150 mM NaCl, 50 mM Tris-HCl [pH 7.0]), dialyzed, and subjected to affinity chromatography using 2 ml of preequilibrated 50% (vol/vol) Strep-Tactin–Sepharose (IBA BioTAGnology). Bound proteins were eluted in four 1-ml fractions with 2.5 mM desthiobiotin in column buffer. Recombinant LcrV_S228_ (rLcrV_S228_) was purified from cell extracts of *E. coli* DH5α as previously described (13). For anti-GSH (ViroGen) immunoblots, LcrV_S228_ was separated by native PAGE. For Edman degradation, purified LcrV_S228_ was separated by SDS-PAGE, electrotransferred to polyvinylidene difluoride (PVDF) membrane, stained with Coomassie blue, excised, and subjected to Edman degradation on an ABI Procise 494 sequencer.

For GST-Sepharose purification, overnight *Y. pestis* cultures were diluted 1:20 into 60 ml of M9-Ca minimal medium, incubated at 26°C for 2 h, and then incubated at 37°C for 3 h to induce type III secretion. Bacteria were sedimented by centrifugation at 7,500 × *g* for 10 min, and 50 ml of supernatant was filtered (0.22-µm-pore membrane). Protein in the filtrate was concentrated in an Amicon Ultra-15 centrifugal device (molecular weight cutoff [MWCO], 10,000) by serial centrifugations at 3,000 × *g* for 10 min and then washed twice with 14 ml of GST-tag column buffer (140 mM NaCl, 40 mM phosphate buffer [pH 7.3]). After the second wash, concentrated sample was transferred to an Eppendorf tube containing 100 μl of preequilibrated 50% (vol/vol) GST-Sepharose (Pierce); the final reaction volume was brought to 1 ml by the addition of GST-tag column buffer. Following overnight incubation at 4°C on a rotating shaker, the sample was centrifuged at 5,000 × *g* for 5 min. The supernatant was discarded, and GST-Sepharose was washed with 1 ml of GST-tag column buffer. After four washes, GST-Sepharose was suspended in 60 μl of 2× reducing SDS-PAGE sample buffer to elute bound proteins. Samples were boiled at 100°C for 5 min, centrifuged at 15,000 × *g* for 5 min, and analyzed by SDS-PAGE and immunoblotting with LcrV-specific antisera.

To purify LcrV_S228_ from infected macrophages, J774.A1 cells were propagated to confluence in 225-cm^2^ tissue culture flasks with 40 ml Dulbecco’s modified Eagle’s medium (DMEM) supplemented with 10% fetal bovine serum and 1% GlutaMAX. Prior to infection, the growth media were exchanged with DMEM supplemented with 1 mM IPTG. Overnight *Y. pestis* p*lcrV*_*S228*_ cultures were diluted 1:20 into 20 ml of HIB and grown at 26°C for 2 h. After 1 mM IPTG supplementation, cultures were propagated at 37°C for 1 h. Bacteria were added at a multiplicity of infection (MOI) of 10 to J774.A1 macrophages. After 3 h of infection at 37°C with 5% CO_2_, tissue culture media were decanted and retained for cytokine analysis; macrophages were suspended in 20 ml of column buffer (150 mM NaCl, 50 mM Tris-HCl [pH 7.0]). Macrophage samples were freeze-thawed twice at −80°C, lysed in a French press at 15,000 lb/in^3^, and centrifuged at 30,000 × *g* for 30 min. The supernatant was subjected to affinity chromatography using 2 ml of preequilibrated 50% (vol/vol) Strep-Tactin–Sepharose. Columns were washed twice with 15 ml of column buffer, and bound proteins were eluted in four 0.5-ml fractions with 5 mM desthiobiotin in column buffer. Eluate was analyzed by silver- and Coomassie-stained SDS-PAGE. Mass spectrometry of trypsin-digested peptides was used to identify proteins. Samples were also analyzed by immunoblotting with antibodies specific for LcrV, RPS3 (Abcam, Inc.), and actin (Sigma); where indicated, the intensities of immunoreactive signals were quantified by densitometry using ImageJ (version v1.49t). Using commercially available enzyme-linked immunosorbent assay (ELISA) kits (eBioscience), the concentrations of IL-1β and IL-18 in the tissue culture media overlying *Y. pestis*-infected J774.A1 macrophages were determined according to the manufacturer’s protocols.

### Mass spectrometry.

Combined liquid chromatography-electrospray ionization mass spectrometry with fraction collection (LC-ESI-MS^+^) was used to analyze protein samples. Sample volumes—typically 400 to 1,000 μl, each containing 0.4 to 0.6 mg protein/ml in 50 mM Tris-HCl (pH 7.0)–150 mM NaCl buffer—were dried in a vacuum concentrator and redissolved in water (250 to 1,000 μl), and aliquots (typically 250 μl) were loaded onto a polymeric reverse-phase column (Agilent PLRP-S; 300-Å pore size, 5-μm particle size, 2.1 by 150 mm), equilibrated in solvent A (water-formic acid, 100/0.1 [vol/vol]), and eluted (100 μl/min) with a linearly increasing concentration of solvent B (acetonitrile-isopropanol-formic acid, 50/50/0.1 [vol/vol/vol]: min/% B, 0/5, 5/5, 45/90, 50/5, and 60/5). The column effluent was passed through a stream splitter, with a proportion (approximately 50%) directed to a fraction collector (1 min/fraction) and the remainder to an Ionspray source connected to a triple-quadrupole mass spectrometer (ABI Sciex API III^+^) scanning in the positive-ion MS mode from *m/z* 500 to 1,850 (step size, 0.3 Da; dwell, 1 ms; 4.82 s/scan; orifice, 90 V). Multiple injections of the same samples were collected into the same fraction collector tubes. For disulfide reduction, protein samples were treated with 6 M guanidine-HCl containing 300 mM dithiothreitol (DTT) for 60 min at room temperature prior to injection onto the liquid chromatograph. Data were collected using software supplied by the instrument’s manufacturer (Tune 2.5-FPU) and were interrogated using MacSpec (version 3.3) for molecular mass calculations from multiply charged ion clusters and BioMultiView (version 1.3.1) for display of the deconvoluted spectra. Protein and peptide molecular weight calculations from known amino acid sequences were made with ExPASy at http://www.expasy.org/proteomics using Compute Pi/MW. For trypsin digestion, proteins of interest were pooled in preweighed glass test tubes and taken to dryness in a vacuum concentrator. An estimation of the protein content of each dried sample was made from the increased mass of the tube after drying. Dried samples were dissolved in 50 mM ammonium bicarbonate, and aliquots (4.34 nmol) were treated with sequencing-grade trypsin (Promega [50 pmol]) in a total volume of 150 μl of 50 mM ammonium bicarbonate at 37°C for 15 h. Aliquots (2 to 10 µl) were injected onto a Phenomenex Kinetex C_18_ column (100-Å pore size, 1.7-µm particle size, 150 by 2.1 mm), equilibrated in solvent A, and eluted (100 μl/min) with a linearly increasing concentration of solvent B (min/% B, 0/3, 2.5/3, 62.5/80, 65/3, and 80/3). The column effluent was directed to an Agilent Jet Stream source connected to a hybrid quadrupole time-of-flight mass spectrometer (Agilent 6550 iFunnel QTOF) scanning in the positive-ion data-dependent mode (*m/z* 200 to 2,500 for MS and 100 to 2,000 for tandem MS [MS/MS]; 4 spectra/s; MS/MS on multiply charged precursors of intensity >/1,000 counts excluded after one cycle; fragmentor, 380 V; collision energy automatically adjusted by instrument manufacturer-supplied software). Data were interrogated using MassHunter software. To reduce disulfide bonds, trypsin-digested samples were treated with 143 mM DTT in 50 mM ammonium bicarbonate at 37°C for 60 min. For the prediction of peptide fragmentation patterns (displays of b and y ions) from a known amino acid sequence, the MS-Product subroutine at Protein Prospector was used (http://prospector.ucsf.edu).

For MALDI-TOF MS analysis, purified LcrV_S228_ was concentrated in HPLC-grade H_2_O. Samples, either treated with DTT or left untreated, were spotted undiluted with sinapinic acid onto a MALDI plate and subjected to matrix-assisted laser desorption ionization–time of flight mass spectrometry (MALDI-TOF MS) using an Autoflex Speed Bruker MALDI instrument in linear mode (10- to 60-kDa range), with myoglobin as a calibration standard (2,000 shots/s at 95% intensity). Integrated data processing software (Bruker) was used to determine the molecular weight of each sample.

### Type III secretion assays.

Assays for *Y. pestis* type III secretion of Yops into the culture medium or injection into HeLa cells, as well as measurements for F1 assembly, were performed as previously described (16, 19). For type III secretion assays, the intensities of immunoreactive signals were quantified by densitometry and analyzed with ImageJ. For murine J774.A1 macrophage infection experiments, 10^5^ cells were seeded on the day prior to infection in 24-well tissue culture dishes in DMEM supplemented with 10% fetal bovine serum (FBS) and 1% GlutaMAX. Prior to infection, growth media were exchanged for DMEM. Overnight *Y. pestis* cultures were diluted 1:20 into 4 ml of HIB, propagated at 26°C for 2 h and then at 37°C for 1 h, added at an MOI of 5 to the J774.A1 cells, and incubated at 37°C with 5% CO_2_. Macrophages were washed once with phosphate-buffered saline (PBS) and stained with 2 µg/ml propidium iodide (Sigma) and 15 µg/ml Hoechst 33342 (Life Technologies, Inc.) for 10 min at room temperature, washed twice in PBS, fixed with 3.7% formaldehyde for 10 min, washed once with PBS, and then visualized by fluorescence microscopy using an Olympus IX81 inverted microscope. Infections were performed in triplicate, and the total numbers of live and dead macrophages were enumerated for three randomly selected fields of view.

To measure *Y. pestis* type III injection into primary neutrophils, Na_2_EDTA-anticoagulated venous blood from healthy human volunteers was layered 1:1 (vol/vol) onto PolymorphPrep and centrifuged at 500 × *g* for 35 min at 20°C. Neutrophils were removed, mixed 1:1 (vol/vol) with 0.5 × Hanks’ balanced saline solution (HBSS), and centrifuged at 350 × *g* for 10 min. Sedimented neutrophils were suspended in 10 ml HBSS, centrifuged at 350 × *g* for 10 min, and suspended in 2 ml of red blood cell lysis buffer (Roche). Samples were centrifuged at 250 × *g* for 5 min, and neutrophils were washed in 10 ml HBSS and suspended in 2 ml RPMI 1640 (Cellgro 10-040-CV) supplemented with 2% human serum albumin. Neutrophil viability (>95%) and purity (>97%) were determined by Trypan blue exclusion and Wright/Giemsa staining, respectively, with at least 400 cells counted per assay. Overnight *Y. pestis* cultures were diluted 1:40 into fresh HIB supplemented with 20 µg/ml chloramphenicol, incubated for 1.5 h at 26°C to reach the mid-exponential phase, and then incubated for 1.5 h at 37°C. Human neutrophils were suspended in Eppendorf tubes at a density of 5.5 × 10^5^ cells in 500 µl of growth medium (RPMI 1640 supplemented with 2% human serum albumin [HSA]). Neutrophils were infected for 3 h (37°C, 5% CO_2_) at an MOI of 10 with *Y. pestis* strains expressing either YopM-Bla (pMM83) or, as a negative control, GST-Bla (pMM91) in a final volume of 600 µl growth medium. At the conclusion of the infection, polymorphonuclear leukocytes (PMNs) were sedimented by centrifugation at 1,500 × *g* for 3 min and suspended in 100 µl of growth medium supplemented with 50 µg/ml kanamycin to abrogate *Y. pestis* growth and type III injection. Neutrophils were stained with 1× CFF2-AM (Invitrogen) for 1 h at room temperature in the dark. Cells were collected by centrifugation at 1,500 × *g* for 3 min, washed once with 500 µl HBSS, and suspended in 500 µl of HBSS Flow (1× HBSS, 0.5 mM EDTA, 25 mM HEPES, 2% bovine serum albumin [BSA] [pH 7.4]). To discriminate between live and dead cells, propidium iodide (PI) was added to each sample at a final concentration of 0.5 µg/ml immediately prior to fluorescence-activated cell sorter (FACS) analysis. A BD FACSCanto flow cytometer was used to analyze at least 10,000 cells per sample, and data were analyzed using FlowJo (v10.0.7) and GraphPad Prism 6. Samples were first gated with the forward and side scatter for the population of singlet PMNs and subsequently gated for live cells by negative PI selection. PI-negative cells were then analyzed for blue fluorescence indicative of *Y. pestis* type III injection of YopM-Bla. Uninfected neutrophils were used to determine the background level of blue fluorescence.

### Animal experiments.

BALB/c mice (*n =* 20) and Brown Norway rats (*n =* 20) were purchased from Charles River Laboratories, Inc. (6- to 8-week old females), and infected by subcutaneous injection with 100 µl PBS with *Y. pestis* strain CO92, TD1, or DE1 at a dose of 20 CFU (mice) or 500 CFU (rats), as previously described (44). At 72 h postchallenge in rats, or when animals (mice and rats) were deemed moribund (laterally recumbent and lethargic with rapid respiration), the animals were euthanized by compressed CO_2_ inhalation. At day 14 postinfection, Brown Norway rats that survived the initial challenge were euthanized as detailed above. Cardiac puncture was performed to obtain sera for analysis of IgG antibody titers. Draining lymph nodes and spleens were removed during necropsy and analyzed for bacterial load by CFU enumeration. Antibody titers were determined by ELISA as previously described (39).

### HPLC analysis of thiol-mBBr derivatives.

*Y. pestis* strains were propagated overnight at 26°C in TMH medium, washed once, subcultured into 4 ml of fresh TMH medium at an initial optical density at 600 nm (OD_600_) of 0.5, and then grown for 5 h at 26°C to reach stationary phase. After transfer of 1 ml of stationary-phase culture to a preweighed Eppendorf tube, *Y. pestis* cells (~200 mg) were sedimented by centrifugation at 7,500 × *g* for 5 min and washed once with 1 ml of 1× PBS. For acid extraction of nonprotein thiols, washed bacterial cells were suspended in 1 ml of extraction buffer (6.3 mM DTPA [diethylenetriaminepentaacetic acid], 0.1% [vol/vol] trifluoroacetic acid [TFA]) and subjected to three rounds of freeze-thawing. Cellular debris was sedimented by centrifugation at 15,000 × *g* for 10 min. The supernatant was stored at −80°C, and the pellet was vacuum-dried at 30°C. The dry weight of the cell pellet was used to normalize the abundance of low-molecular-weight (LMW) thiols present in the corresponding supernatant fraction. A similar approach was used to analyze LMW thiol abundance in *Y. pestis* growth media (LB broth and TMH medium). Specifically, 1 ml of extraction buffer was added to an Eppendorf tube containing 250 µl of growth medium, and the sample was subjected to three freeze-thaw cycles as described above. After the sample was centrifuged at 15,000 × *g* for 10 min to pellet any insoluble material, the supernatant fraction was immediately collected, transferred to another Eppendorf tube, and stored at −80°C. For monobromobimane (mBBr) derivatization, 615 μl of HEPPS {3-[4-(2-hydroxyethyl)piperazinyl]propanesulfonic acid} buffer (200 mM HEPPS, 6.3 mM DTPA [pH 8.2]) was mixed with 25 μl of 20 mM TCEP, a nonthiol disulfide reductant. To this mixture, 250 μl of a thiol standard mixture or sample extract was added, followed by the addition of 10 μl of 0.5 mM *N*-acetyl cysteine, which served as an internal control. After preincubation of this reaction mixture in a heat block at 45°C for 10 min, derivatization was carried out by adding 10 μl of 50 mM mBBR and incubating the samples in the dark in a heat block at 45°C for 30 min. The reaction was terminated by the addition of 100 μl of 1 M methanesulfonic acid, samples were passed through 0.45-μm-pore nylon syringe filters (Pall-Gelman Labs) and stored at −20°C. HPLC analysis of thiol-mBBr derivatives was performed over a 250- by 4.6-mm Thermo Scientific Hypersil Gold column with a 5-μm particle-size guard column (Thermo Scientific). The column temperature was maintained at 40°C, and the injection volume was 10 μl. Separation of LMW thiol-mBBr adducts was achieved by using a linear gradient in which the mobile phase was supplemented with 0.1% TFA. The gradient was derived from buffer A (water, 10% acetonitrile, 0.1% TFA) and buffer B (acetonitrile, 0.1% TFA), with a continuous flow rate of 1 ml per min and the following parameters: 0 to 5 min, 0% buffer B; 5 to 16.2 min, linear gradient from 0 to 10.6% buffer B; 16.2 to 29.8 min, linear gradient from 10.6 to 21.1% buffer B; 29.9 to 39.9 min, 100% buffer B; 40 to 45 min, 0% buffer B. The elution profile was monitored by fluorescence, using an excitation wavelength of 394 nm and an emission wavelength of 490 nm.

### *Y. pestis* survival in vertebrate blood and serum.

*Y. pestis* strains were propagated overnight in 4 ml LB broth at 26°C. After 0.5 ml of *Y. pestis* overnight culture was centrifuged at 7,500 × *g* for 5 min, sedimented bacteria were washed with 1 ml of 1× PBS and suspended in 1 ml of 1× PBS. After dilution of the bacterial suspension at 1:20 in 1× PBS, its OD_600_ was measured in order to calculate the volume required to inoculate 10^5^ CFU of the bacterial suspension into 4 ml of sheep blood or serum (Hemostat Laboratories). Cultures were incubated for 18 h at 26 or 37°C. To enumerate the fold change in viable *Y. pestis* cells over 18 h of growth, CFU counts were determined both pre- and postincubation by serially diluting sheep blood or sheep serum cultures and plating on LB agar.

### Statistics.

Statistical analysis was performed using Prism 6 (GraphPad Software, Inc.). For *in vitro* experiments, the two-tailed unpaired Student’s *t* test was used to compare differences between two groups, and one-way analysis of variance (ANOVA) with Tukey’s posttest was used to compare differences between more than two groups. For *in vivo* experiments, differences between two groups were analyzed using either the Mann-Whitney *U* test (CFU) or the two-tailed unpaired Student’s *t* test (serum IgG titers). Survival analysis of *Y. pestis*-infected animals was performed using the Gehan-Breslow-Wilcoxon test. A *P* value of <0.05 was determined to be statistically significant.
