# Supplementary figures and images for: Glutathionylation of Yersinia pestis LcrV and Its Effects on Plague Pathogenesis
Source: mBio. 2017 May 16;8(3):e00646-17. doi: 10.1128/mBio.00646-17 (PMC5433101; doi:10.1128/mBio.00646-17)

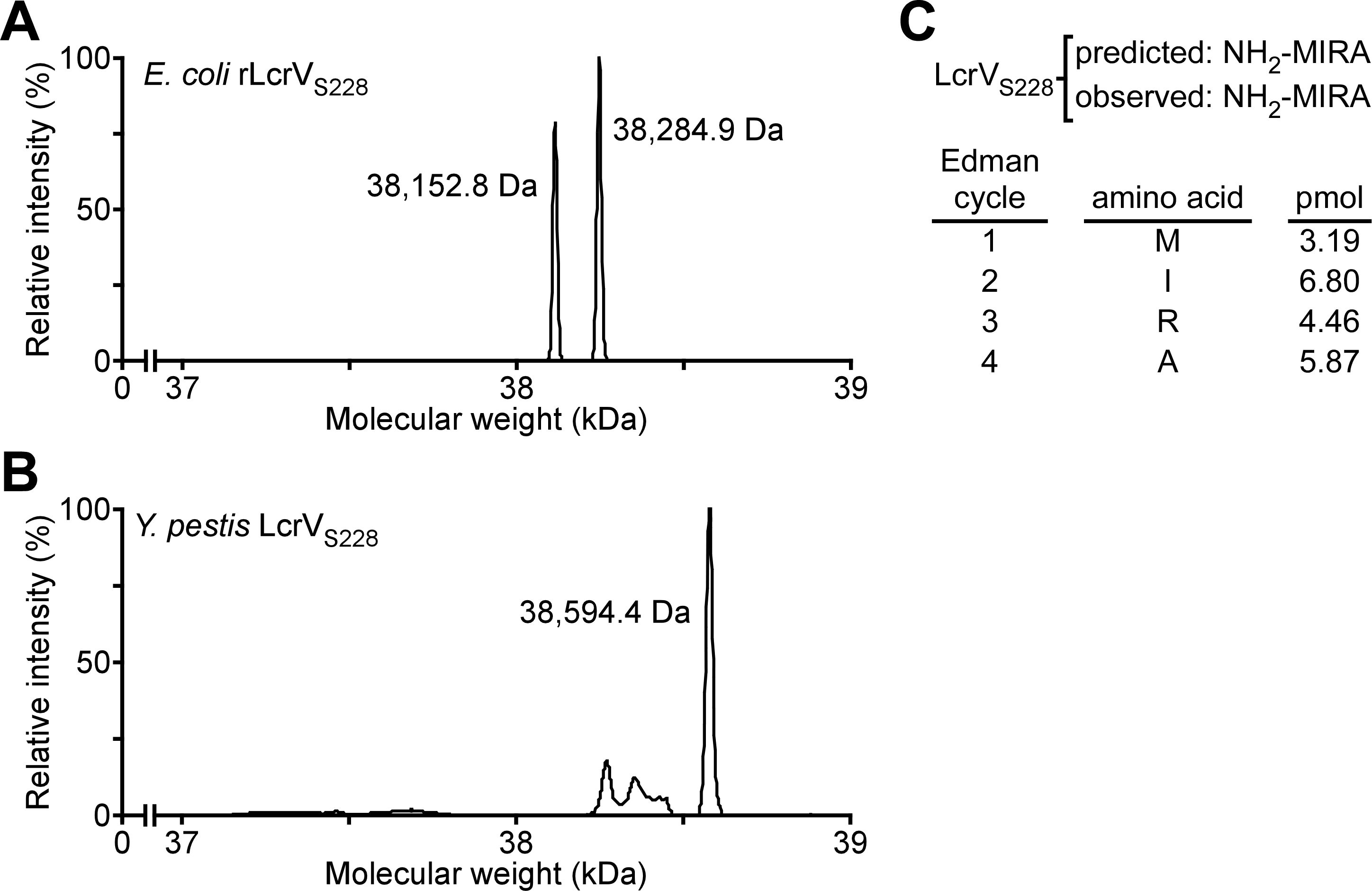

Supplement: FIG S1 [file mbo003173312sf1.tif]

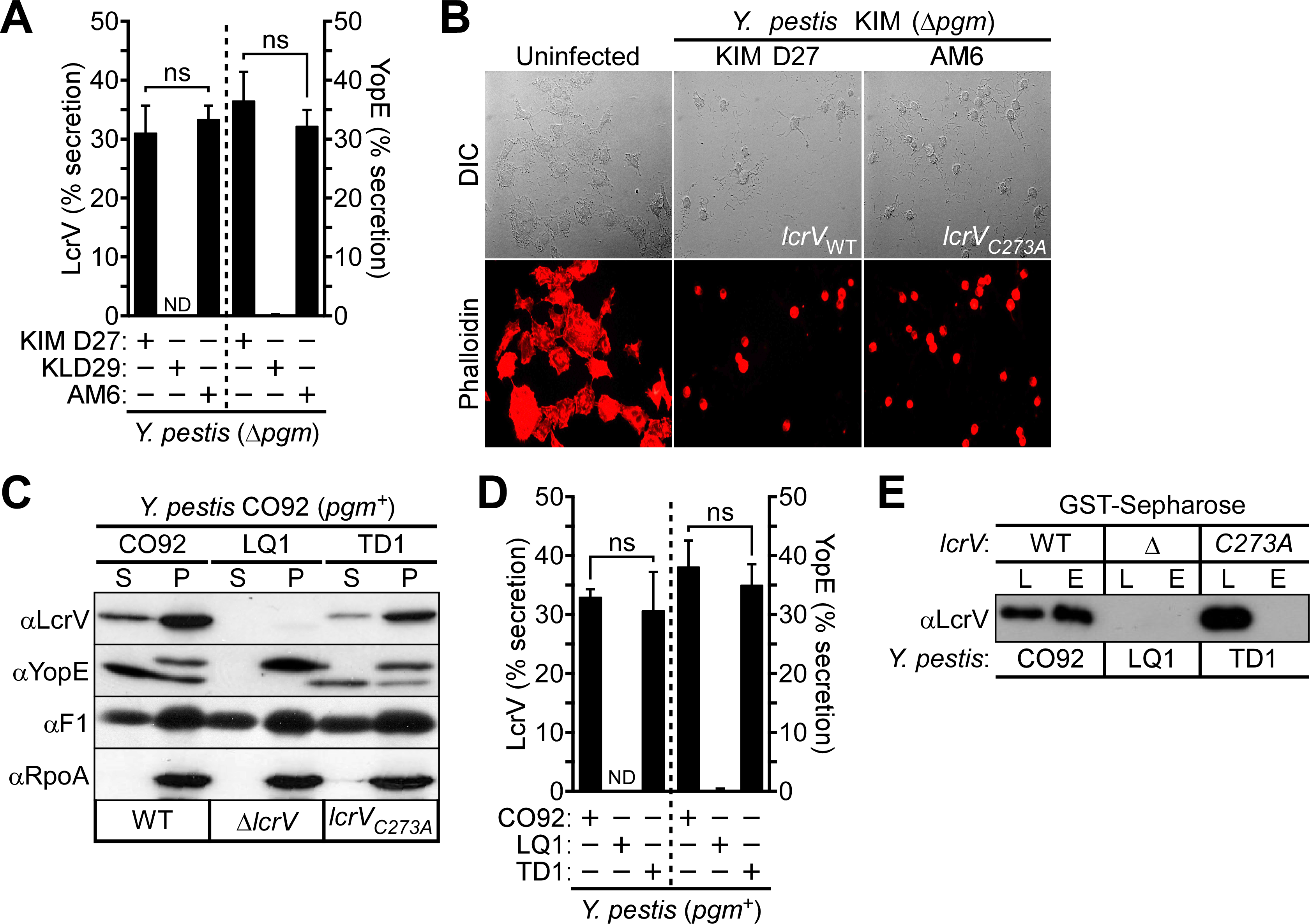

Supplement: FIG S2 [file mbo003173312sf2.tif]

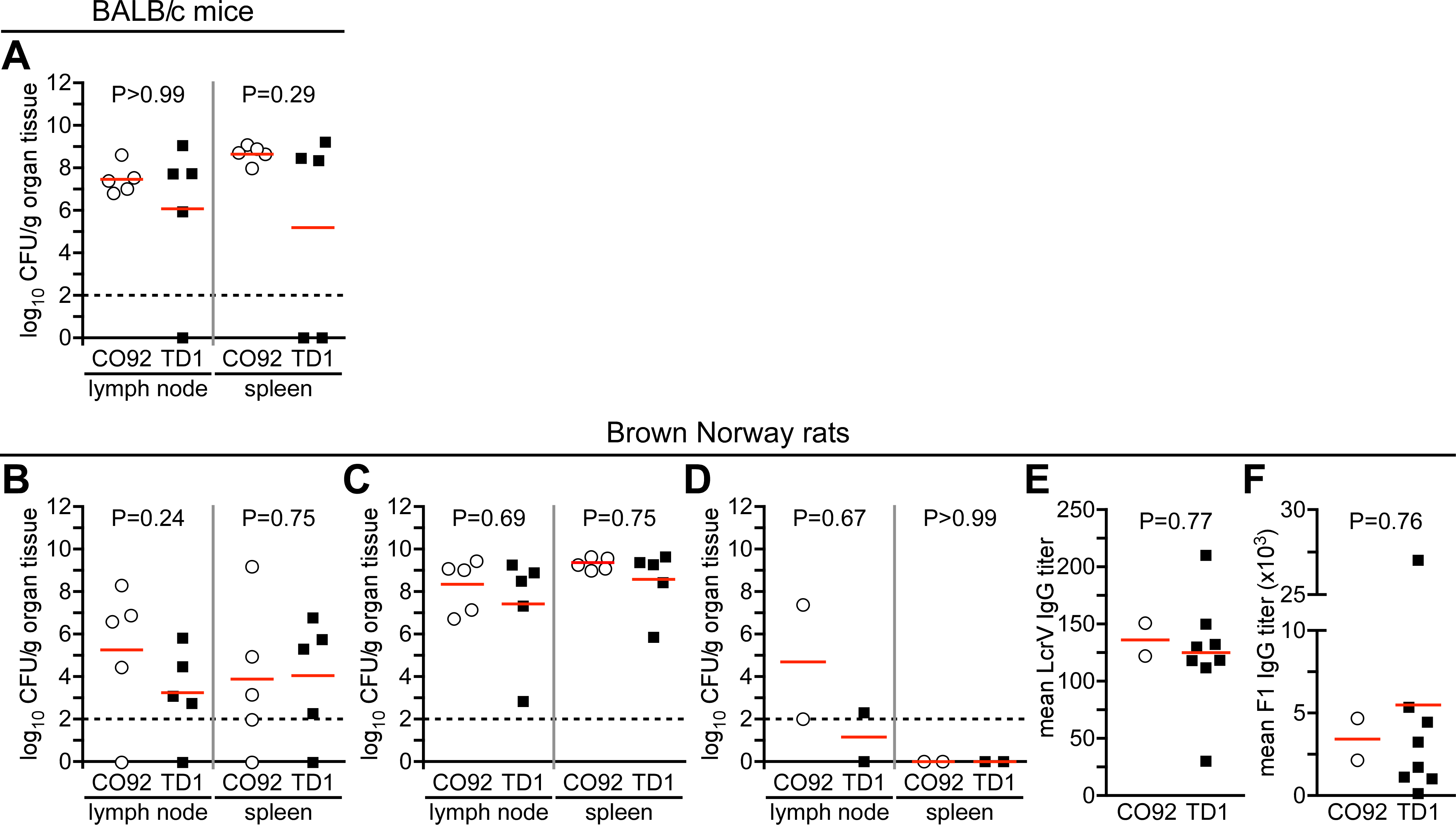

Supplement: FIG S3 [file mbo003173312sf3.tif]

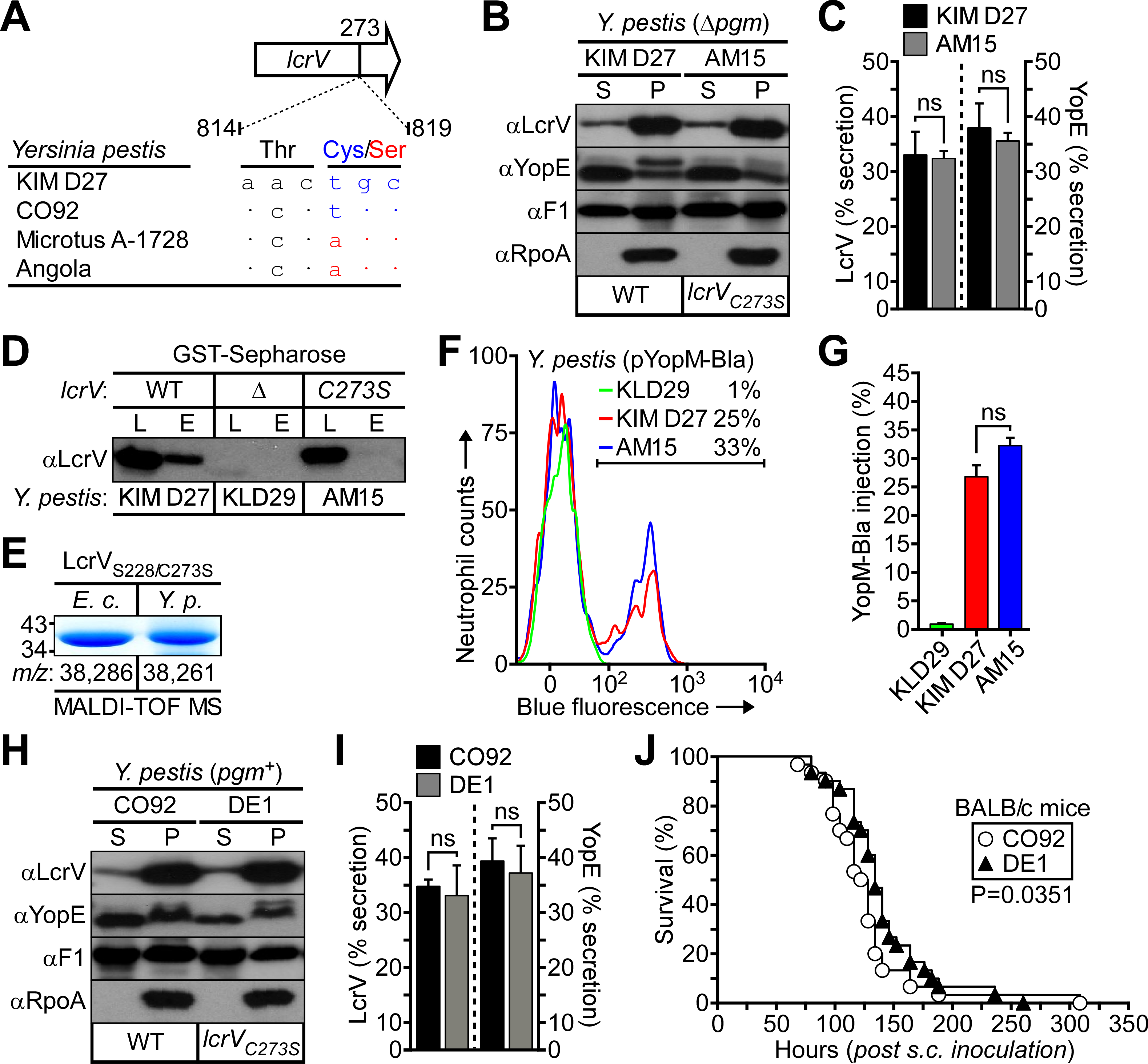

Supplement: FIG S4 [file mbo003173312sf4.tif]

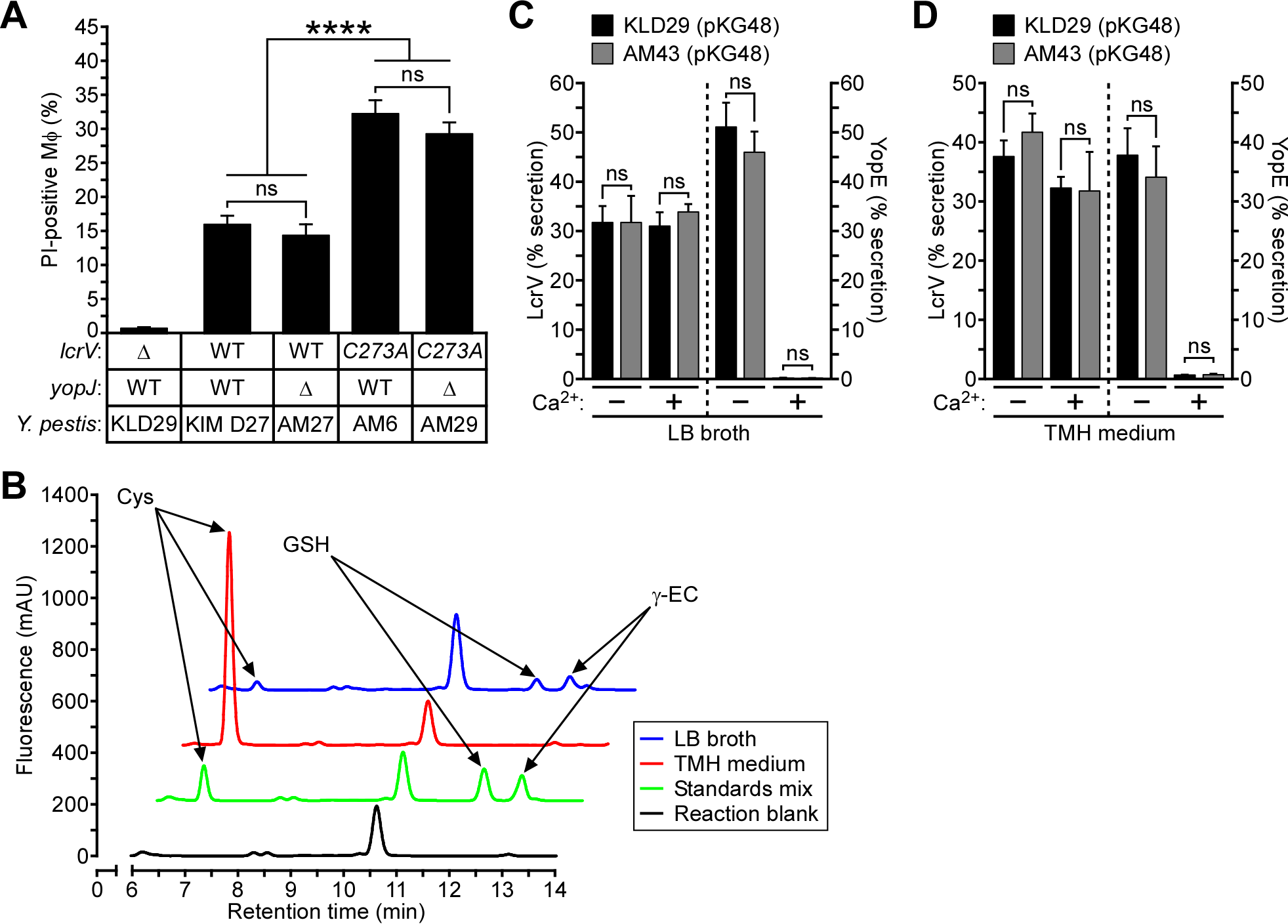

Supplement: FIG S5 [file mbo003173312sf5.tif]
