# Supplementary material for: Glutathionylation of Yersinia pestis LcrV and Its Effects on Plague Pathogenesis
Source: mBio. 2017 May 16;8(3):e00646-17. doi: 10.1128/mBio.00646-17 (PMC5433101; doi:10.1128/mBio.00646-17)
Supplement: TABLE S1 [file mbo003173312st1.docx]

| **Table S1. Summary of mass spectrometry analysis of tryptic peptides from *Y. pestis* LcrV_S228_ and *E. coli* rLcrV_S228_** | | | | | |
| --- | --- | --- | --- | --- | --- |
| **LcrV_S228_ Peptide^a^** | **Calculated Mass (Da)^b^** | **rLcrV_S228_^c^** | **Delta^d^** | **LcrV_S228_^e^** | **Delta^f^** |
| 21-49^g^ | 3192.66 | NF^i^ | – | 3193.37 | -0.71 |
| 215-236 | 2612.98 | NF | – | 2612.18 | 0.80 |
| 1-20 | 2516.86 | 2516.18 | 0.68 | NF | – |
| 219-239 | 2473.74 | 2473.13 | 0.61 | NF | – |
| 19-40 | 2420.79 | 2420.38 | 0.41 | 2421.48 | -0.69 |
| 117-137 | 2409.80 | 2409.68 | 0.12 | 2410.13 | -0.33 |
| 21-42 | 2408.73 | 2409.68 | -0.95 | NF | – |
| 316-334 | 2244.55 | NF | – | 2243.58 | 0.97 |
| 296-315 | 2237.45 | NF | – | 2238.02 | -0.57 |
| 21-40 | 2165.47 | 2165.63 | -0.16 | 2165.93 | -0.46 |
| 219-236 | 2129.37 | 2129.33 | 0.04 | 2129.63 | -0.26 |
| 101-116 | 1963.18 | 1963.28 | -0.10 | 1963.58 | -0.40 |
| 4-18 | 1861.00 | 1861.43 | -0.43 | 1860.68 | 0.32 |
| 103-116 | 1735.87 | NF | – | 1736.48 | -0.61 |
| 87-100 | 1599.73 | 1599.08 | 0.65 | NF | – |
| 117-130 | 1596.88 | 1596.28 | 0.60 | 1596.88 | 0.00 |
| 50-62 | 1596.72 | NF | – | 1596.88 | -0.16 |
| 153-165 | 1514.74 | 1513.98 | 0.76 | 1514.78 | -0.04 |
| 74-86 | 1505.82 | 1505.39 | 0.43 | 1506.78 | -0.96 |
| 197-208 | 1491.62 | 1491.44 | 0.18 | 1491.89 | -0.27 |
| 138-150 | 1450.59 | 1450.66 | -0.07 | 1450.38 | 0.21 |
| 241-253 | 1449.67 | 1450.28 | -0.61 | 1450.58 | -0.91 |
| 87-99 | 1443.54 | 1443.89 | -0.35 | 1444.38 | -0.84 |
| 177-189 | 1408.53 | 1408.38 | 0.15 | 1408.78 | -0.25 |
| 63-73 | 1286.53 | 1285.88 | 0.65 | 1286.68 | -0.15 |
| 166-176 | 1277.48 | 1276.94 | 0.54 | 1277.39 | 0.09 |
| 285-295 | 1256.42 | 1255.94 | 0.48 | 1256.69 | -0.27 |
| 155-165 | 1245.39 | 1244.99 | 0.40 | 1245.14 | 0.25 |
| 63-72 | 1157.65 | 1157.99 | -0.34 | 1157.99 | -0.34 |
| 306-315 | 1133.58 | 1133.99 | -0.41 | 1134.14 | -0.56 |
| 296-305 | 1120.57 | 1120.94 | -0.37 | 1120.94 | -0.37 |
| 54-62 | 1064.53 | NF | – | 1064.99 | -0.46 |
| 246-254 | 1064.53 | NF | – | 1064.99 | -0.46 |
| 41-49 | 1044.58 | 1044.74 | -0.16 | 1044.89 | -0.31 |
| 55-62 | 936.43 | 936.59 | -0.16 | 936.74 | -0.31 |
| 246-253 | 908.42 | 908.54 | -0.12 | 908.99 | -0.57 |
| 320-326 | 897.40 | 897.74 | -0.34 | 897.74 | -0.34 |
| 263-269 | 874.38 | 874.49 | -0.11 | 874.79 | -0.41 |
| 327-334 | 847.43 | 847.58 | -0.15 | 847.79 | -0.36 |
| 131-137 | 830.44 | 830.54 | -0.10 | 830.99 | -0.55 |
| 190-196 | 819.42 | 819.59 | -0.17 | 819.74 | -0.32 |
| 43-49 | 801.46 | 801.59 | -0.13 | 801.74 | -0.28 |
| 255-262 | 772.44 | 772.79 | -0.35 | 772.79 | -0.35 |
| 240-245 | 686.47 | 686.84 | -0.37 | 686.84 | -0.37 |
| 209-214 | 667.32 | 668.30 | -0.98 | 667.79 | -0.47 |
| 241-245 | 558.37 | 558.59 | -0.22 | 558.59 | -0.22 |
| 50-53 | 549.25 | 549.59 | -0.34 | 549.59 | -0.34 |
| 316-319 | 534.32 | 534.59 | -0.27 | 534.29 | 0.03 |
| 151-154 | 502.32 | NF | – | 501.59 | 0.73 |
| 215-218 | 501.32 | 501.29 | 0.03 | 501.59 | -0.27 |
| 1-3 | 418.24 | 418.34 | -0.10 | 418.34 | -0.10 |
| 270-284 | 1681.75 | 1680.74 | 1.01 | NF | – |
| 270-284 -GSH | 1987.07 | NF | – | 1987.43 | -0.36 |
| 270-284^h^ | 1680.70 | 1680.71 | -0.0083 | NF | – |
| 270-284-GSH^h,j^ | 1985.79 | NF | – | 1985.79 | -0.0002 |
| ^a,b^Peptide sequences derived from *in silico* tryptic cleavage of LcrV_S228_ and their calculated masses  ^c,d^Observed mass of tryptic peptides derived from rLcrV_S228_ that had been purified by affinity chromatography from cell lysates of *E. coli* DH5α (pKG48) and then identified by liquid chromatography/electrospray ionization mass spectrometry (LC/ESI-MS) and their delta with the predicted mass  ^e,f^Observed mass of tryptic peptides derived from LcrV_S228_ that had been purified by affinity chromatography from culture supernatants of *Y. pestis* KLD29 (pKG48) and then identified by LC/ESI-MS and their delta with the predicted mass  ^g^This data set was obtained with the Sciex API III^+^ instrument; the calculated masses are a mixture of monoistopic and average depending on the resolution observed in the spectra unless otherwise indicated  ^h^These data were obtained with the Agilent QTOF instrument; the calculated masses are monisotopic  ^i^NF=not found  ^j^270-284-GSH=glutathionylated peptide 270-284 | | | | | |
