# Supplementary material for: Glutathionylation of Yersinia pestis LcrV and Its Effects on Plague Pathogenesis
Source: mBio. 2017 May 16;8(3):e00646-17. doi: 10.1128/mBio.00646-17 (PMC5433101; doi:10.1128/mBio.00646-17)
Supplement: TABLE S2 [file mbo003173312st2.docx]

| **Table S2. Tandem mass spectrometry of the 1985.79 Da LcrV_S228_ peptide** | | | | | | | |
| --- | --- | --- | --- | --- | --- | --- | --- |
| **Residue** | **#** | **Calculated**  ***m/z*** | **Found^a^**  ***m/z*** | **Error**  **(ppm)** | **+305.068159**  **calculated** | **Found**  ***m/z*** | **Error**  **(ppm)** |
|  | | | | ***y ions*** | | | |
| D | 15 | – |  |  |  |  |  |
| N | 14 | 1566.6853 | NF | – | 1871.753459^b^ | NF^c^ |  |
| N | 13 | 1452.6424 | NF | – | 1757.710559 | 1757.68243 | 16.00 |
| E | 12 | 1338.5994 | NF | – | 1643.667559 | 1643.66496 | 1.58 |
| L | 11 | 1209.5568 | NF | – | 1514.624959 | 1514.62361 | 0.89 |
| S | 10 | 1096.4728 | NF | – | 1401.540959 | 1401.53408 | 4.91 |
| H | 9 | 1009.4408 | NF | – | 1314.508959 | 1314.50293 | 4.59 |
| F | 8 | 872.3818 | 872.36486 | -19.4 | 1177.449959 | 1177.44546 | 3.82 |
| A | 7 | 725.3134 | NF | – | 1030.381559 | 1030.37738 | 4.06 |
| T | 6 | 654.2763 | NF | – | 959.344459 | 959.34008 | 4.56 |
| T | 5 | 553.2286 | NF | – | 858.296759 | 858.296 | 0.88 |
| C | 4 | 452.1810 | NF | – | 757.249159 | 757.25393 | -6.30 |
| S | 3 | 349.1718 | 349.17224 | 1.3 | 654.239959 | NF | – |
| D | 2 | 262.1397 | 262.14044 | 2.8 | 567.207859 | NF | – |
| K | 1 | 147.1128 | 147.11296 | 1.1 | 452.180959 | NF | – |
|  | | | | ***b ions*** | | | |
| D | 1 | – |  |  |  |  |  |
| N | 2 | 230.0771 | 230.07836 | 5.48 | 535.145259^d^ | NF | – |
| N | 3 | 344.1201 | 344.12084 | 2.15 | 649.188259 | NF | – |
| E | 4 | 473.1627 | 473.16257 | -0.27 | 778.230859 | NF | – |
| L | 5 | 586.2467 | 586.24761 | 1.55 | 891.314859 | NF | – |
| S | 6 | 673.2788 | 673.27996 | 1.72 | 978.346959 | NF | – |
| H | 7 | 810.3377 | 810.33583 | -2.31 | 1115.405859 | NF | – |
| F | 8 | 957.4061 | 957.40085 | -5.48 | 1262.474259 | NF | – |
| A | 9 | 1028.4432 | 1028.44074 | -2.39 | 1333.511359 | NF | – |
| T | 10 | 1129.4909 | 1129.48625 | -4.12 | 1434.559059 | NF | – |
| T | 11 | 1230.5386 | 1230.52721 | -9.26 | 1535.606759 | NF | – |
| C | 12 | 1333.5477 | NF | – | 1638.615859 | NF | – |
| S | 13 | 1420.5798 | NF | – | 1725.647959 | NF | – |
| D | 14 | 1535.6067 | NF | – | 1840.674859 | NF | – |
| K | 15 | – |  |  |  |  |  |
| ^a^Collisionally-induced-dissociation of the 1985.79 Da peptide in a tandem mass spectrometry experiment  ^b^y+305.068159  ^c^NF=not found  ^d^b+305.068159 | | | | | | | |
