# Supplementary material for: Glutathionylation of Yersinia pestis LcrV and Its Effects on Plague Pathogenesis
Source: mBio. 2017 May 16;8(3):e00646-17. doi: 10.1128/mBio.00646-17 (PMC5433101; doi:10.1128/mBio.00646-17)
Supplement: TABLE S3 [file mbo003173312st3.docx]

| **Table S3. Peptide mass fingerprinting by LC/MS/MS identifies macrophage RPS3 as a ligand of translocated LcrV^a^** | | | | |
| --- | --- | --- | --- | --- |
| **Observed *m/z*^b^** | **Calculated *m/z*^c^** | **Δ*m/z*^d^** | **Matching RPS3 Peptide^e^** | **Amino Acid Residues** |
| 1024.5822 | 1024.5826 | -0.0004 | KFVADGIFK | 10-18 |
| 896.4876 | 896.4876 | 0.0000 | FVADGIFK | 11-18 |
| 1092.5688 | 1092.5684 | 0.0004 | AELNEFLTR | 19-27 |
| 1423.6714 | 1423.6700 | 0.0014 | ELAEDGYSGVEVR | 28-40 |
| 1029.6315 | 1029.6303 | 0.0012 | TEIIILATR | 46-54 |
| 1156.7048 | 1156.7048 | 0.0000 | IRELTAVVQK | 66-75 |
| 1728.8584 | 1728.8592 | -0.0008 | RFGFPEGSVELYAEK | 76-90 |
| 1572.7597 | 1572.7581 | 0.0016 | FGFPEGSVELYAEK | 77-90 |
| 1302.6839 | 1302.6834 | 0.0005 | GLCAIAQAESLR | 95-106 |
| 798.5195 | 798.5196 | -0.0001 | LLGGLAVR | 109-116 |
| 2468.1941* | 2468.1915 | 0.0026 | FVDGLMIHSGDPVNYYVDTAVR | 152-173 |
| 714.4507 | 714.4509 | -0.0002 | QGVLGIK | 179-185 |
| 1143.5858 | 1143.5867 | -0.0009 | IMLPWDPSGK | 188-197 |
| 1458.8314 | 1458.8315 | -0.0001 | KPLPDHVSIVEPK | 202-214 |
| 2910.5853 | 2910.5823 | 0.0030 | KPLPDHVSIVEPKDEILPTTPISEQK | 202-227 |
| 1470.7697 | 1470.7686 | 0.0011 | DEILPTTPISEQK | 215-227 |
| 1573.8052* | 1573.8043 | 0.0009 | GGKPEPPAMPQPVPTA | 228-243 |
| ^a^Peptide mass fingerprinting was performed by the Taplin Biological Mass Spectrometry Facility (Harvard Medical School) by bottom-up proteomics using using micro-capillary liquid chromatography—tandem mass spectrometry (LC/MS/MS)  ^b^An asterisk (*) denotes that the observed ion was accompanied by a less intense ion with an increased *m/z* (+15.99); this accompanying ion was interpreted as an oxygen adduct of the parent ion resulting from oxidation of the peptide's single methionine residue  ^c^Monoisotopic *m/z* calculations were determined using Protein Prospector (http://prospector.ucsf.edu/prospector/mshome.htm)  ^d^Observed *m/z* - Calculated *m/z*  ^e^SWISS-PROT database searches were used to identify matching peptides in the 40S ribosomal protein S3 (RPS3) of *Mus musculus* (http://www.uniprot.org/uniprot/P62908) | | | | |
