# Supplementary material for: Glutathionylation of Yersinia pestis LcrV and Its Effects on Plague Pathogenesis
Source: mBio. 2017 May 16;8(3):e00646-17. doi: 10.1128/mBio.00646-17 (PMC5433101; doi:10.1128/mBio.00646-17)
Supplement: TABLE S4 [file mbo003173312st4.docx]

| **Table S4. MALDI-TOF MS analysis of LcrV_S228_ and LcrV_S228/C273S_ purified from *Y. pestis* supernatants or *E. coli* extracts^a^** | | | | | | | | |
| --- | --- | --- | --- | --- | --- | --- | --- | --- |
| **Protein** | **Strain^b^** | **Genotype** | **Molecular weight (Da)** | | | | | **Predicted Structure** |
|  |  |  | **Calculated^c^ (±GSH)** | **Observed**  **-DTT** | **Δ^d^**  **rLcrV_S228_** | **Δ^e^**  **LcrV_S228_** | **Observed^f^**  **+DTT** |  |
| rLcrV_S228_ | *E. coli* DH5α  (pKG48) | WT  (p*lcrV_S228_*) | 38,280.22 | 38,271.66 | -8.56 | -313.88 | 38,281.05 | [LcrV_S228_]-SH |
| LcrV_S228_ | *Y. pestis* KLD29  (pKG48) | Δ*lcrV*  (p*lcrV_S228_*) | 38,585.54 | 38,583.10 | 302.88 | -2.44 | 38,282.55 | [LcrV_S228_]-GSH |
| LcrV_S228_ | *Y. pestis* AM43  (pKG48) | Δ*lcrV*, Δ*gshB*  (p*lcrV_S228_*) | – | 38,592.16 | 311.94 | 6.62 | 38,281.27 | [LcrV_S228_]-GSH |
| rLcrV_S228/C273S_ | *E. coli* DH5α  (pAM199) | WT  (p*lcrV_S228/C273S_*) | 38,264.16 | 38,286.09 | 21.93 | – | – | [LcrV_S228/C273S_]-SH |
| LcrV_S228/C273S_ | *Y. pestis* KLD29  (pAM199) | Δ*lcrV*  (p*lcrV_S228/C273S_*) | 38,264.16 | 38,261.34 | -2.82 | – | – | [LcrV_S228/C273S_]-SH |
| ^a^Purified LcrV was either left untreated (-DTT) or treated with dithiothreitol (+DTT), and then spotted undiluted with sinapinic acid onto a MALDI plate and analyzed by matrix-assisted laser desorption ionization/time-of-flight mass spectrometry (MALDI-TOF MS)  ^b^All bacterial strains were propagated in LB broth during Strep-Tactin affinity purification experiments  ^c^Average molecular weights of non-glutathionylated rLcrV_S228_ (38,280.22 Da) and glutathionylated LcrV_S228_ (38,585.54 Da), as well as non-glutathionylated rLcrV_S228/C273S_ and LcrV_S228/C273S_ (38,264.16 Da), were calculated using Protein Prospector (http://prospector.ucsf.edu/prospector/mshome.htm)  ^d^Observed molecular weight – Calculated molecular weight of rLcrV_S228_ or rLcrV_S228/C273S_  ^e^Observed molecular weight – Calculated molecular weight of glutathionylated LcrV_S228_  ^f^DTT treatment of *Y. pestis* LcrV_S228_ collapses the molecular weight to match that of *E. coli* rLcrV_S228_ | | | | | | | | |
