# Supplementary material for: Glutathionylation of Yersinia pestis LcrV and Its Effects on Plague Pathogenesis
Source: mBio. 2017 May 16;8(3):e00646-17. doi: 10.1128/mBio.00646-17 (PMC5433101; doi:10.1128/mBio.00646-17)
Supplement: TABLE S5 [file mbo003173312st5.docx]

| **Table S5. Bacterial strains, plasmids and primers used in this study** | | | |
| --- | --- | --- | --- |
| **Strains** | | **Description** | **Reference** |
| ***E. coli*** | | | |
| DH5α | | supE44 ΔlacU169 (ϕ80lacZΔM15) hsdR17 recA1 endA1 gyrA96 thi1 relA1 | (40) |
| S17-1 | | thi pro recA::RP4-2-Tc::Mu-Km::Tn7 str (λpir^+^), hsdR mutant, hsdM mutant | (41) |
| ***Y. pestis* KIM (Δ*pgm*)** | | | |
| KIM D27 | | Wild-type isolate (Δ*pgm*, pCD1^+^, pFra^+^, pPCP1^+^); also called KIM5 | (18) |
| KLD29 | | Δ*lcrV* | (18) |
| AM6 | | *lcrV_C273A_* | This study |
| AM15 | | *lcrV_C273S_* | This study |
| AM27 | | Δ*yopJ* | This study |
| AM29 | | *lcrV_C273A_*, Δ*yopJ* | This study |
| AM43 | | Δ*lcrV*, Δ*gshB* | This study |
| ***Y. pestis* CO92 (*pgm^+^*)** | | | |
| CO92 | | Wild-type isolate (*pgm*^+^, pCD1^+^, pFra^+^, pPCP1^+^) | (18) |
| LQ1 | | *ΔlcrV* | (19) |
| TD1 | | *lcrV_C273A_* | This study |
| DE1 | | *lcrV_C273S_* | This study |
|  | | | |
| **Plasmids** | | **Description^a,b^** | **Reference** |
| pLC28 | | Suicide vector, Cm^r^ | (42) |
| pCVD442 | | Suicide vector, Amp^r^ | (43) |
| pNM77 | | pHSG575 derivative, *tac* promoter fused to wild-type *lcrV*, Cm^r^ | (16) |
| pKG48 | | pNM77 derivative, *tac* promoter fused to *lcrV_S228_* (Strep-tag inserted after LcrV residue 228), Cm^r^ | (13) |
| pAM128 | | pKG48 derivative, *tac* promoter fused to *lcrV_S228/C273A_*, Cm^r^ | This study |
| pAM199 | | pKG48 derivative, *tac* promoter fused to *lcrV_S228/C273S_*, Cm^r^ | This study |
| pYopM-Bla (pMM83) | | pHSG576 derivative, native *yopM* promoter fused to *yopM-bla*, Cm^r^ | (8) |
| pAM105 | | pLC28 derivative, *lcrV_C273A_* allelic exchange construct for *Y. pestis* KIM D27, Cm^r^ | This study |
| pCVD442-lcrV_C273A_ | | pCVD442 derivative, *lcrV_C273A_* allelic exchange construct for *Y. pestis* CO92, Amp^r^ | This study |
| pAM155 | | pCVD442 derivative, *lcrV_C273S_* allelic exchange construct, Amp^r^ | This study |
| pAM178 | | pCVD442 derivative, *yopJ* deletion construct, Amp^r^ | This study |
| pAM196 | | pCVD442 derivative, *gshB* deletion construct, Amp^r^ | This study |
|  | | | |
| **Primers** | **Sequence (5′→3′)^c,d^** | | |
| P140 for | CACTTTGCCACCACCgcgTCGGATAAGTCCAGG | | |
| P140 rev | CCTGGACTTATCCGAcgcGGTGGTGGCAAAGTG | | |
| P141 for | G**TCTAGA**GATATCGGCTTAACGCCTG | | |
| P141 rev | C**GGATCC**AACTAGCTTACCTAACTC | | |
| P142 for | AGC**GTCGAC**GATATCGGCTTAACGCCTG | | |
| P142 rev | AGC**GAGCTC**AACTAGCTTACCTAACTC | | |
| P190 for | CACTTTGCCACCACCaGCTCGGATAAGTCCA | | |
| P190 rev | TGGACTTATCCGAGCtGGTGGTGGCAAAGTG | | |
| P227 for | AAA**GAGCTC**CGGGAGCTATCTGATTTGCTTAG | | |
| P227 rev | AAA**AAGCTT**TTATTTATCCTTATTCAGGGAATTAACAGC | | |
| P228 for | AAA**AAGCTT**TGTATTTTGGAAATCTTGCTCCAG | | |
| P228 rev | AAA**GTCGAC**GGAACAGATGGTAATACTGTAACCGAAC | | |
| P296 for | AAA**GAGCTC**GGATTGAATGGGAACATGTGGTTC | | |
| P296 rev | AAA**GGATCC**TCTTTTCTCCTAGTGGGAAATGGGGT | | |
| P297 for | AAA**GGATCC**TACATAAAACGGGGTGTGGTGTT | | |
| P297 rev | AAA**GTCGAC**GCAACAGTCACTTTTGTCATGAGAA | | |
| ^a^Chloramphenicol-resistant (Cm^r^)  ^b^Ampicillin-resistant (Amp^r^)  ^c^Boldface nucleotides indicate a restriction site  ^d^Lowercase nucleotides differ from the wild-type sequence and result in an amino acid substitution | | | |
